# SARS−CoV−2 spike S1-mediated HIF−2α activation in retinal endothelial cells suggests a mechanism contributing to post−COVID endothelial dysfunction

**DOI:** 10.3389/fimmu.2026.1770758

**Published:** 2026-03-09

**Authors:** Andrea Ribeiro, Timon Wallraven, Maciej Lech, Kristina Adorjan, Hans Christian Stubbe, Martina Seifert, Anna Wöhnl, Veronika Kesseler, Johanna Negele, Christoph Schmaderer

**Affiliations:** 1 School of Medicine, Klinikum Rechts Der Isar, Department of Nephrology, Technical University of Munich, Munich, Germany; 2 Medizinische Klinik Und Poliklinik IV, LMU University Hospital Munich, Munich, Germany; 3 Department of Psychiatry and Psychotherapy, University of Bern, Bern, Switzerland; 4 Institute for Medical Immunology, Charité-Universitätsmedizin Berlin, Corporate Member of Freie Universität Berlin and Humboldt Universität zu Berlin, Berlin, Germany; 5 BCRT-Berlin Institute of Health (BIH) Center for Regenerative Therapies, Charité-Universitätsmedizin Berlin, Berlin, Germany; 6 German Center for Infection Research (DZIF), Partner Site Munich, Munich, Germany

**Keywords:** angiogenesis, endothelial dysfunction, HIF-2α, HIF-2α inhibition, hypoxia, long COVID, post-COVID syndrome

## Abstract

**Background:**

Post-COVID-19 syndrome (PCS) is characterized by persistent symptoms such as fatigue, cardiovascular abnormalities, and cognitive impairment. Endothelial dysfunction (ED) has been proposed as a contributing factor, but underlying mechanisms remain unclear. We investigated whether SARS-CoV-2 spike protein subunit 1 (S1) is sufficient to induce ED in human retinal endothelial cells (HRECs) *in vitro* and whether pharmacologic inhibition of HIF-2α signaling modulates endothelial barrier integrity.

**Methods:**

In this study, we characterized 41 PCS patients and 24 pre-pandemic healthy controls. The effects of recombinant S1 and plasma from patients with severe PCS on endothelial function were assessed in HRECs. Belzutifan was used as a pharmacologic probe to assess the role of HIF-2α signaling in S1- and plasma-associated endothelial responses.

**Results:**

PCS patients exhibited elevated erythropoietin, VEGF, and MCP-1 levels compared with controls. VEGF correlated with anti-S1 IgG and was upregulated at the mRNA level in S1-exposed HRECs. Additionally, *in vitro* exposure to S1 induced ROS production, transient HIF-1α and sustained HIF-2α activation, VEGFR2 upregulation, and impaired endothelial barrier integrity. Plasma from patients with severe PCS increased ROS production and induced modest alterations in endothelial barrier function in HRECs. In both S1- and PCS-plasma–treated cells, pharmacologic HIF-2α inhibition with belzutifan improved endothelial barrier integrity.

**Conclusion:**

These findings identify a spike-responsive, HIF-2α–associated ED pathway in retinal endothelial cells. Modulation of this pathway altered endothelial barrier responses to both recombinant S1 and plasma from patients with PCS, highlighting a candidate mechanism that may contribute to PCS-associated vascular dysfunction.

## 1 Introduction

Post-COVID-19 syndrome (PCS), also known as long COVID, affects 10 to 30% of individuals after acute SARS-CoV-2 infection and is characterized by a range of symptoms, including fatigue, shortness of breath, chest tightness, cough, headache, cardiovascular abnormalities, and cognitive dysfunction, which can persist for weeks, months, or even years (1–6). The exact causes of PCS are still uncertain, with hypotheses including virus or viral remnant persistence, which may contribute to prolonged immune activation (7, 8); autoimmunity, supported by the presence of autoantibodies targeting receptors involved in vascular and immune regulation (8–11); microbiota disturbances, which can influence systemic inflammation and immune homeostasis (8, 12); reactivation of dormant infections unrelated to SARS-CoV-2, potentially triggering inflammatory cascades and immune dysregulation (13, 14); chronic inflammation-induced tissue damage, which may drive long-term organ dysfunction (15); and ongoing cardiovascular effects potentially driven by microthrombi and persistent vascular dysfunction (16, 17). Although these factors arise from distinct mechanisms, they all have the potential to promote chronic inflammation and oxidative stress, and disrupt vascular homeostasis, ultimately converging on endothelial dysfunction (ED). In turn, ED can exacerbate immune dysregulation and inflammation, creating a self-perpetuating cycle that sustains PCS pathophysiology and may contribute to the persistence of symptoms (10, 18–21).

SARS-CoV-2 can damage endothelial cells (ECs) directly through the angiotensin-converting enzyme-2 (ACE2) receptor or indirectly through cytokine storm resulting from the immune response to viral replication, leading to oxidative stress, reduced nitric oxide (NO) availability, and endothelial barrier disruption (22–25). These processes can cause vascular endotheliitis, marked by inflammation of the endothelial lining. Endotheliitis promotes ED through a cytokine surge, driving systemic immune dysregulation, thrombosis, and widespread organ dysfunction (26–30). Evidence suggests that viral replication can persist for months in various tissues, potentially serving as reservoirs for spike proteins (31–33). Spike proteins may persist in the body for up to 12 months, sustaining chronic inflammation and immune activation (7, 34). The resulting environment, characterized by elevated reactive oxygen species (ROS), can activate hypoxia-inducible factors (HIFs), which play an important role in aggravating the immune response and inflammation during both the acute and chronic phases of SARS-CoV-2 infection (35–37). The two major HIFs, HIF-1α and HIF-2α, are transcription factors that regulate key physiological processes, including metabolism, cell proliferation, and angiogenesis, and impact endothelial function through the expression of vascular endothelial growth factor (VEGF) (38–40). VEGF acts on specific tyrosine kinase receptors, the VEGFR1─3, initiating the assembly of the vasculature. Among them, VEGFR2 is mainly distributed in vascular endothelial cells and acts as a major signal transducer for angiogenesis (41).

To experimentally interrogate the contribution of this pathway to endothelial stress responses, pharmacologic interference with HIF-2α represents one possible approach. Belzutifan (MK-6482; formerly PT2977) is a highly selective small-molecule inhibitor reported to selectively disrupt HIF-2α transcriptional activity and has been characterized primarily in systems with genetically driven HIF-2α activation. *In vitro*, such compounds can be used as pathway probes to assess whether observed cellular responses are consistent with HIF-2α–dependent signaling (42–45).

In this study, we tested whether exposure to the SARS-CoV-2 spike protein subunit 1 (S1) is sufficient to activate endothelial stress pathways *in vitro*, with a focus on HIF signaling. Using primary human retinal endothelial cells (HRECs), we examined whether S1 can engage HIF-dependent responses associated with ED and whether pharmacologic inhibition of HIF-2α modulates these effects. This approach explores a candidate mechanism that could contribute to endothelial alterations observed in PCS.

## 2 Material and methods

### 2.1 Experimental model and study participant details

This study is part of the “All Eyes on PCS” study, which is an observational, single-center study investigating the underlying causes of PCS. The study protocol with a detailed description of the methods has previously been published (46). It has been written following the Strengthening the Reporting of Observational studies in Epidemiology (STROBE) guidelines (47). A standardized questionnaire on the day of recruitment, detailed clinical evaluations, and patient-reported outcome measures were used to assess the PCS severity score (48) and the COVID-19 Yorkshire Rehabilitation Scale (C19‐YRS) (49). The study was conducted in accordance with the Declaration of Helsinki and approved by the local ethics committee (Ethics Committee of the Technical University of Munich, School of Medicine, Klinikum rechts der Isar; Approval number: 2022-317-S-SR), and was previously registered (https://clinicaltrials.gov/ct2/show/NCT05635552). All participants in this study provided written informed consent.

### 2.2 Laboratory values

Blood sampling was performed as previously described (50). Standard laboratory measurements were performed in an ISO-certified routine 8 laboratory. VEGF, interleukin-6 (IL-6), intercellular adhesion molecule 1 (ICAM-1), vascular cell adhesion molecule 1 (VCAM-1), monocyte chemoattractant protein-1 (MCP-1), C-X-C motif chemokine ligand 10 (CXCL10), and regulated on activation, normal T-cell expressed and secreted (RANTES) in the patient`s plasma was quantified using the Cytometric Bead Array Flex system (BD Biosciences, Cat. No.558336, 558276, 560269, 560427, 558287, 558280, 558324 respectively) according to the instructions of the manufacturer. Samples were acquired on a FACSCanto II flow cytometer (BD Biosciences), and sample data analysis was performed with FCAP Array Software (BD Biosciences). SARS-CoV-2 Spike RBD IgG levels in plasma were measured by enzyme-limited immunosorbent assay (ELISA) using a Legend Max ELISA Kit (BioLegend, Cat. No. 447707) following the manufacturer’s recommendations. Erythropoietin (EPO) was measured using a Quantitative IVD ELISA (R&D Systems, Cat. No. DEP00) under the manufacturer’s instructions. Absorbances were measured at 450 nm in a Multiskan FC microplate reader (Thermo Scientific).

### 2.3 Cell culture and *in vitro* experiments

Primary human retinal endothelial cells (HRECs) were purchased from Innoprot and cultured in endothelial basal medium supplemented with 5% fetal bovine serum, 1% endothelial cell growth supplement, and 1% penicillin/streptomycin solution (all from Innoprot). They were maintained at 37 °C and 5% CO_2_ in a humidified incubator and used at passages 3-6, after which they were discarded. To model sustained spike protein–induced endothelial signaling, cells were treated with active recombinant human coronavirus SARS-CoV-2 Spike glycoprotein S1 (S1) (Abcam) at 100 ng/mL, or mock-treated in the presence or absence of 50 nM belzutifan (MedchemExpress). The chosen S1 concentration was selected based on prior endothelial studies demonstrating robust phenotypic responses at nanomolar concentrations (51, 52). Although this exceeds median plasma levels reported in PCS, similar concentrations have been observed in subsets of patients and may reflect localized or repeated endothelial exposure (53). Accordingly, this dose was used to probe chronic endothelial stress mechanisms rather than to directly mimic circulating plasma concentrations. HIF protein stabilization was achieved using 100 µM cobalt chloride (CoCl_2_), as described (54). Lipopolysaccharide (LPS-EB, InvivoGen) was used at 100 ng/mL where indicated. For plasma exposure experiments, cells were grown in endothelial basal medium containing 5% FBS, 1% endothelial cell growth supplement, and 1% penicillin-streptomycin. On the experiment day, the FBS was replaced with 2% human plasma (HC or PCS) while retaining 1% endothelial cell growth supplement to maintain cell viability. The 2% plasma concentration was selected based on prior studies demonstrating that this level minimizes cytotoxicity while preserving biologically relevant inter-group differences (55–57). 3 IU/mL of heparin was added to plasma samples to prevent coagulation upon contact with endothelial monolayers.

### 2.4 RNA extraction, reverse transcription and quantitative real-time PCR

Total RNA was extracted from HRECs using the Norgen Total RNA Purification Kit (Norgen Biotek), following the manufacturer’s protocol. cDNA was synthesized from 1 μg of total RNA by quantitative real-time reverse transcription polymerase chain reaction (RT-qPCR) using Superscript II reverse transcriptase (Thermo Fisher) as per the manufacturer’s guidelines. RT-qPCR from cDNA was performed in a Light Cycler 480 (Roche). GAPDH mRNA was used as a reference transcript for relative quantification. The 2ΔCT method was used to assess the expression levels of target genes in each sample. ΔCT values were calculated by subtracting the CT value of the target gene from that of the reference gene. The 2ΔCT values were then derived by raising 2 to the power of the ΔCT value, providing the relative expression levels of the target gene in each experimental sample. Fold induction was not applied for comparisons of gene expression within samples. The resulting 2ΔCT values were then subjected to statistical analysis to identify significant differences in gene expression between groups. Controls consisting of ddH2O were negative for the target and reference genes. The melting curve profiles were analyzed. All primers (**Supplementary Table 1**) used for amplification were purchased from Metabion.

### 2.5 Immunofluorescence experiments

HRECs were grown in 8-well chamber slides previously coated with 2 µg/cm2 fibronectin (Innoprot). Once HRECs formed a confluent monolayer, cells were subjected to the desired treatment. After treatment, monolayers of HRECs were fixed in 4% formaldehyde for 15 minutes at room temperature. Cells were then treated with permeabilization buffer containing 0.1% Triton X-100 in PBS for 10 minutes at room temperature, followed by treatment with blocking buffer containing 3% BSA and 0.1% Triton X-100 in PBS for 30 minutes at room temperature. Subsequently, cells were incubated with primary antibodies against NF-κB, HIF-1α, HIF-2α, BNIP-3, GLUT-1, VEGFR2, claudin-5, and VE-cadherin (all from Cell Signaling; Cat. No. 8242, 36169, 71565, 44060, 73015, 2479, 49564, 2500, respectively), or PDK-1 (Abcam, Cat. No. ab202468) in an antibody dilution buffer containing 1% BSA and 0.1% Triton X-100 in PBS, overnight, at 4 °C. Next, cells were washed three times in PBS and incubated with a fluorescently tagged goat anti-rabbit secondary antibody (Cell Signaling, Cat. No. 4412S) in antibody dilution buffer containing 1% BSA in PBS for 1 hour at room temperature protected from light. Cells were then washed three times in PBS and incubated with phalloidin (Abcam, Cat. No. ab176756) diluted in 1% BSA in PBS for 90 minutes. The cells were subsequently washed three times with PBS, and DAPI (Vector Laboratories) was added. Images were acquired with a Leica DM RBE fluorescence microscope. Corrected Total Cell Fluorescence (CTCF) was calculated using the formula: CTCF=Integrated Density−(Area×Mean Fluorescence of Background). All measurements, including the integrated density, cell area, and background fluorescence, were performed using ImageJ. Measurements were taken from all cells within at least 3 fields of each sample to ensure statistical reliability. Background fluorescence was determined by selecting regions without cells in each image as background regions of interest. For F-actin, stress-fiber content was quantified on phalloidin-stained images by converting images to 8-bit, applying a single global intensity threshold (chosen on representative fields and kept constant for all conditions) to generate binary masks, and calculating the percentage of stress-fiber–positive area per field as the area fraction (%Area) of white pixels in ImageJ.

### 2.6 Measurement of ROS

Cellular ROS generation was assessed using DCFDA/H2DCFDA-Cellular ROS assay kit (Abcam, Cat. No. ab113851). ROS in the cells oxidize DCFH, yielding the fluorescent product 2′,7′-dichlorofluorescein (DCF). HRECs were seeded in a dark clear-bottom 96-well microplate (Corning) at a density of 25,000 cells per well and allowed to adhere overnight. Cells were subsequently incubated with DCFDA for 45 minutes in the dark. Following DCFDA removal, cells were exposed to the desired stimuli, and fluorescence intensity was measured at Ex/Em= 485/535 nm at 60-minute intervals over a period of 6 hours. Mitochondrial ROS generation was evaluated using MitoSOX Red Mitochondrial Indicator (Invitrogen) as described (58). HRECs were exposed to the intended treatment for a period of 4 hours. Following treatment, the cells were harvested into flow cytometry tubes and incubated with MitoSOX Red reagent for 20 minutes at 37 °C in a light-protected environment. Following the incubation period, the cells were prepared for flow cytometry analysis, with the quantification of MitoSOX expression being measured in the PE channel.

### 2.7 Measurement of NO

Total NO production in supernatants was assessed using a commercially available fluorometric nitric oxide assay kit (Sigma–Aldrich, Cat. No. 482655) according to the manufacturer’s instructions. The assay is based on the enzymatic conversion of nitrate to nitrite by nitrate reductase, followed by the addition of 2,3-diaminonapthalene (DAN) and NaOH, which converts nitrite to a fluorescent compound. Briefly, 100 µL of supernatant from mock-treated cells or cells treated with S1, HC-plasma or PCS-plasma, each diluted in assay buffer, was dispensed into a 96-well plate. 10 µL of enzyme co-factors and 10 µL of nitrate reductase were added to each well. The plate was incubated at room temperature for 1 hour. After the required incubation period, 10 µL of DAN reagent was added to each well and the plate was incubated for 10 minutes followed by the addition of 20 µL of NaOH to each well. Fluorescence intensity was measured at Ex/Em= 375/415 nm. The nitrate + nitrite concentration was calculated from a nitrate standard curve.

### 2.8 Transendothelial electric resistance (TEER) measurement

The barrier function of confluent endothelial cell monolayers was estimated using electric cell‐substrate impedance sensing (ECIS) model Z-Theta (Applied Biophysics) as described (59). HREC monolayers were grown on standard 8-well arrays (8W10E + PET) (Ibidi). Resistance was measured continuously in a multi-frequency setup. After the resistance at 4,000 Hz reached a stable plateau of >1,000-ohm, HRECs were incubated with fresh medium in the presence or absence of S1 or belzutifan, and the capacity of the barrier integrity disruption was measured over a 72-hour period. In a second approach, plasma samples from HC or PCS patients were diluted to 2% (v/v) in cell culture medium, either in the presence or absence of belzutifan, and tested for a period of 42 hours. After treatment, cells were continuously monitored in real time in multiple frequency modes. Values were normalized to time = 0 for easier comparisons.

### 2.9 Quantification and statistical analyses

Statistical calculations were performed using GraphPad Prism software (v10.3.0) and R Studio (v2024.04.2 + 764). The distribution of variables was verified by the Shapiro–Wilk test for normality. Student’s t-test was used for comparisons between two groups, whereas one-way ANOVA or two-way ANOVA followed by Tukey’s *post hoc* test was used for comparisons among more than two groups for normally distributed data. To compare baseline characteristics, the χ2 test was used for categorical values. For nonnormally distributed data, the Mann–Whitney U test or Kruskal–Wallis test followed by Dunn’s *post hoc* test was used, depending on group size. All values are expressed as the means ± SDs for normally distributed data and as medians with IQRs for nonnormally distributed data. Exploratory correlation analyses were performed using Spearman’s or Pearson’s correlation, as indicated, based on data distribution. Correlation analyses involving plasma biomarkers, disease severity scores, and endothelial functional readouts were conducted using pairwise complete observations without correction for multiple testing and are intended as hypothesis-generating. Correlation heatmaps were generated in R using *ggplot2* and *pheatmap*, with patients ordered by unsupervised hierarchical clustering based on Jaccard distance of binary symptom profiles. TEER slope was calculated for each experiment by fitting a linear regression model to TEER measurements over time (0 to 48h). For experiments assessing the effect of S1 on cells, n refers to independent biological experiments. For patient plasma experiments, each biological replicate represents plasma from a single, unique participant applied to one well per condition. Thus, n reflects independent patient samples rather than technical replicates. Clinical data were assessed and generated by two independent researchers and subsequently checked for differences (double-data verification).

## 3 Results

### 3.1 Main baseline characteristics of the PCS cohort

**Figure 1:**
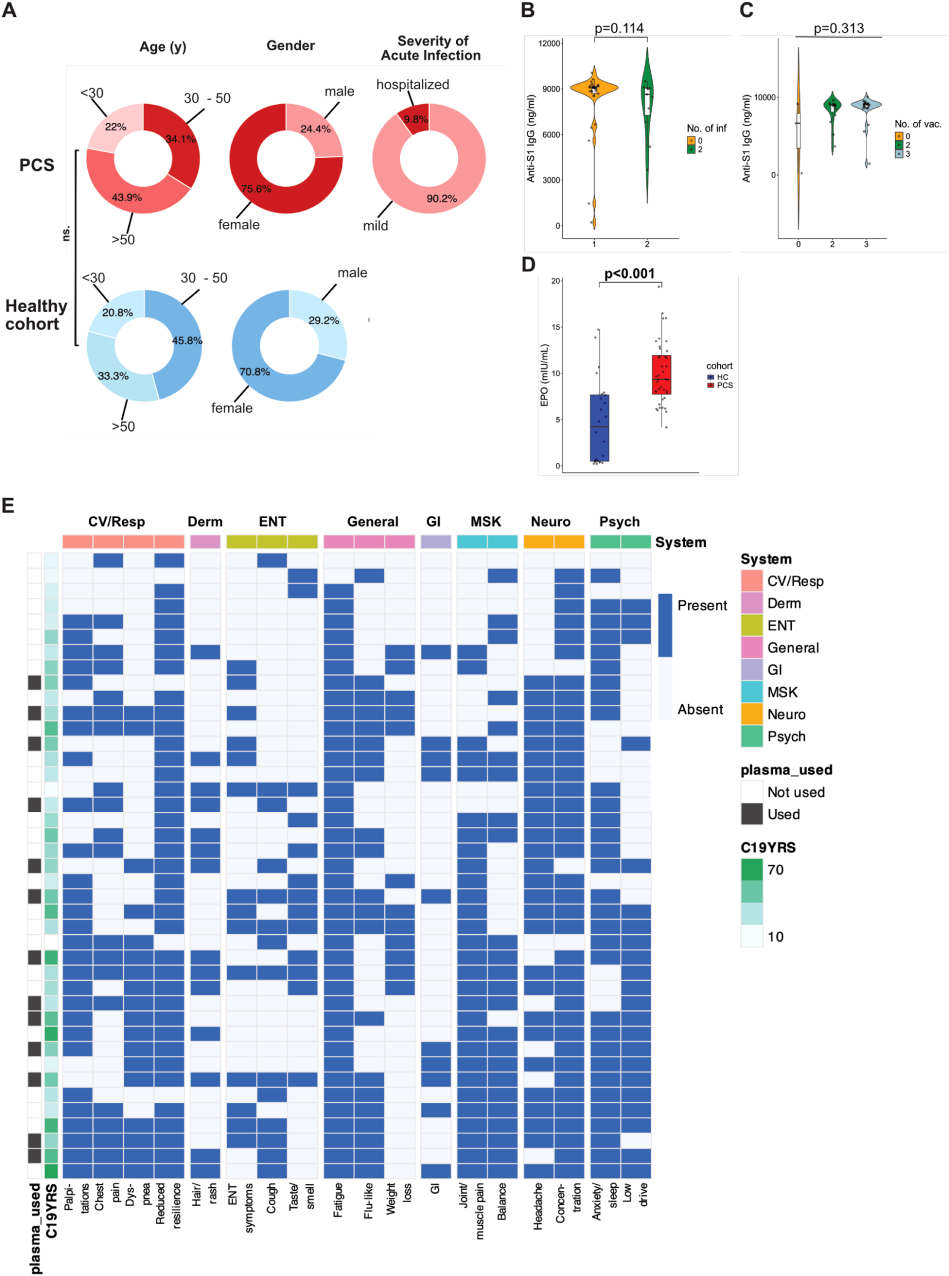
Demographic characteristics, serological features, and symptom distribution in the PCS cohort. **(A)** Distribution of age and sex in PCS patients (n = 41, red color scale) and healthy controls (HC; n = 24, blue color scale), as well as severity of acute SARS-CoV-2 infection among PCS patients. **(B)** Anti-S1 IgG levels according to the number of documented SARS-CoV-2 infections in the PCS cohort. **(C)** Anti-S1 IgG levels according to the number of SARS-CoV-2 vaccinations in the PCS cohort. **(D)** Plasma erythropoietin (EPO) levels in PCS patients compared with healthy controls. **(E)** Patient-by-symptom heatmap illustrating the presence or absence of self-reported PCS symptoms at the individual patient level. Rows represent individual PCS patients and columns indicate symptoms, grouped by organ system. Patients were ordered using unsupervised hierarchical clustering based on Jaccard distance of binary symptom profiles. System group annotations (general, musculoskeletal [MSK], ear-nose-throat [ENT], cardiovascular/respiratory [CV/Resp], neurological [Neuro], dermatological [Derm], gastrointestinal [GI], and psychological [Psych]) are shown above the heatmap. The COVID-19 Yorkshire Rehabilitation Scale (C19-YRS) score is displayed as a continuous row annotation. Patients whose plasma samples were used for *in vitro* endothelial experiments are indicated in the row annotation. P-values were determined using χ² test and Student’s t-test **(A)**, Mann-Whitney U test **(B, E)**, and Kruskal-Wallis test **(C)**.

The study included 41 PCS patients (mean age 42.2 years, ± 12.2, 75.6% female) who had previously been infected with SARS-CoV-2 and exhibited typical symptoms of PCS. Patients were age- and gender-matched with 24 healthy volunteers recruited before the COVID-19 pandemic (mean age 44.6 years, ± 12.2 years, 70.8% female; HC group) (**Figure 1A**). Main baseline characteristics of the cohort are summarized in **Supplementary Table 2**. The prevalence of cardiovascular risk factors, such as arterial hypertension, nicotine abuse, diabetes mellitus, and obesity, was similar between the groups. A total of 73.2% of the PCS patients had one infection, and 26.8% had two infections, with omicron (24.4%) being the most frequent variant. Twenty-three (56.1%) patients were vaccinated three times, 15 (36.6%) patients were vaccinated twice, and 3 (7.3%) patients were not vaccinated. Anti-S1 IgG levels did not vary with the number of infections (**Figure 1B**) or vaccinations (**Figure 1C**), and levels were higher in PCS patients than in HCs (**Supplementary Table 2**). The median duration of PCS was 12.7 months (± 8.4). Among the PCS patients, 19.5% experienced work loss, with a median duration of sick leave of 122.0 days (4.0 to 291.0 days). EPO levels (**Figure 1D**) were significantly higher in PCS patients compared with HCs, while remaining within the established reference range. **Figure 1E** shows a patient-by-symptom heatmap illustrating individual-level symptom co-occurrence in our PCS cohort. Patients were ordered using unsupervised hierarchical clustering, placing individuals with more similar symptom constellations next to one another. The heatmap highlights inter-individual heterogeneity with substantial symptom overlap across patients, including a high frequency of fatigue, reduced physical resilience, and concentration difficulties. While there is a tendency for patients with fewer reported symptoms to appear toward the top of the heatmap and those with broader multisystem involvement toward the bottom, clustering does not segregate patients into clearly distinct organ system-restricted subgroups. Consistent with this, the C19-YRS score (overall mean level: 38.20 [± 18.21]) and pre-defined systems (e.g., cardiovascular/respiratory, neurological, etc.) vary across the clustered patient order, indicating that overall symptom burden and clinical severity are related but not perfectly aligned. To further explore a potential link between dysregulated erythropoiesis, S1 levels, and PCS severity, we correlated hemoglobin (Hb) and hematocrit (Hct) with anti-S1 IgG levels and C19-YRS. Both Hb and Hct showed a non-significant negative correlation with anti-S1 (**Supplementary Figures 1B, C**). After controlling for confounders, low Hb levels were significantly associated with high anti-S1 IgG levels (**Supplementary Table 3**). Both lower levels of Hb (**Supplementary Figure 1D**) and Hct (**Supplementary Figure 1E**) were significantly correlated with higher C19-YRS severity score. The association remained significant for Hct but was weaker for Hb after controlling for potential confounders (**Supplementary Table 3**).

### 3.2 Elevated plasma levels of ED markers in PCS

**Figure 2:**
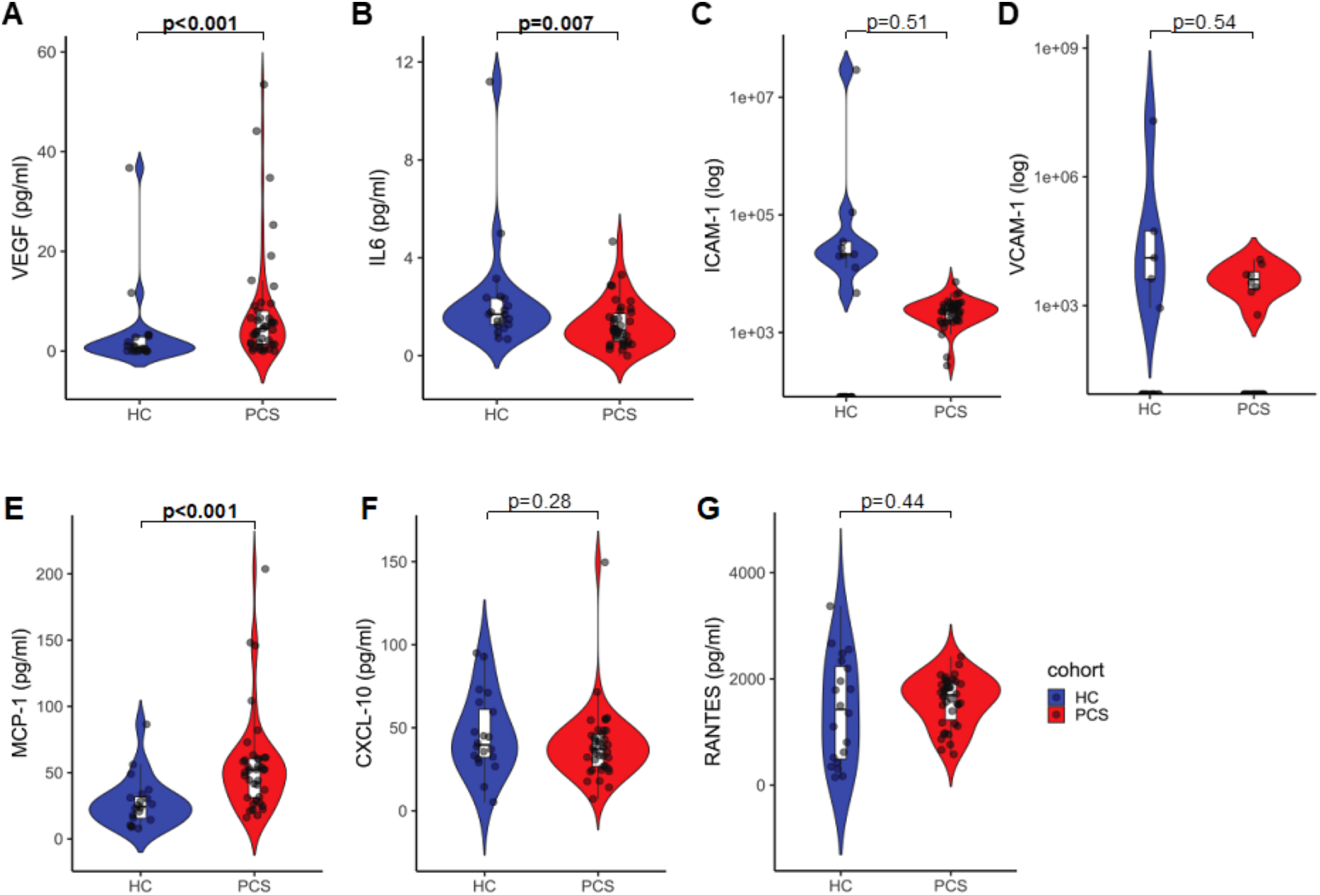
Parameters of inflammation and ED in patients with PCS compared with HCs. Plasma levels of **(A)** VEGF, **(B)** IL-6, **(C)** ICAM-1, **(D)** VCAM-1, **(E)** MCP-1, **(F)** CXCL10, and **(G)** RANTES were measured in PCS patients and healthy cohort (HC) by cytometric bead array. VEGF was analyzed in 41 PCS patients and 20 HC; IL-6, MCP-1, RANTES, and CXCL10 were measured in 38 PCS patients and 20 HC. Plots show values with the mean indicated by a line. Data are shown as means ± SD. A p-value of <0.05 was considered statistically significant. P-values were determined by Mann*–*Whitney U test.

In prior studies, we and others have demonstrated that ED is a hallmark of PCS, potentially linked to chronic inflammation (17, 20, 60) and angiogenic activation (37, 61). Here, we profiled the plasma samples from the participants for a panel of seven markers, including VEGF, IL-6, ICAM-1, VCAM-1, MCP-1, CXCL10, and RANTES. Among the selected markers, VEGF and MCP-1 were significantly higher in PCS patients (**Figure 2**A and 2C). RANTES, ICAM-1, VCAM-1, and CXCL10 did not significantly increase in PCS-plasma compared with HC-plasma (**Figures 2D-G**). Surprisingly, IL-6 levels were lower in plasma samples from PCS patients than in those from HCs. However, both groups presented IL-6 values within the normal range (**Figure 2B**).

Given reports of higher anti-S1 IgG in participants with PCS, we performed exploratory correlations of VEGF and inflammatory markers with anti-S1 IgG levels. Inflammatory markers such as IL-6, RANTES, MCP-1, and CXCL10 were not significantly correlated (data not shown). Higher VEGF was positively correlated with anti-S1 IgG levels (**Supplementary Figure 1A**); however, this effect was weaker after controlling for potential confounders in a multivariate linear model (**Supplementary Table 3**).

### 3.3 S1 induces increased *VEGF* mRNA expression without upregulating immune activation markers in HRECs

**Figure 3:**
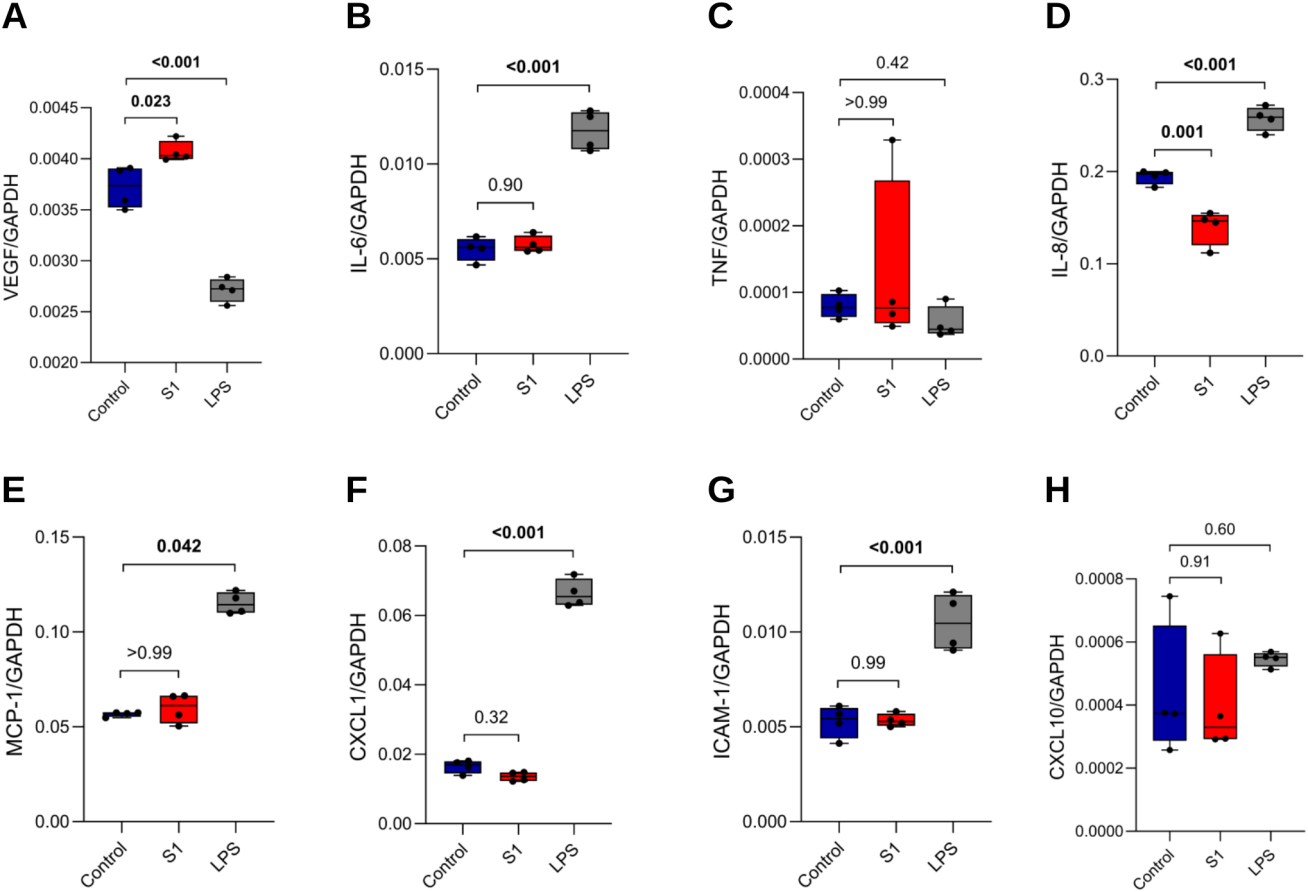
S1 induces *VEGF* mRNA expression in HRECs but not immune activation markers. HRECs were mock-treated (control, n=4), stimulated with S1 (100 ng/mL, n=4), or LPS (100 ng/mL, n=4) for 4 h. mRNA expression of **(A)** *VEGF*, **(B)** *IL-6*, **(C)** *TNF*, **(D)** *IL-8*, **(E)** *MCP-1*, **(F)** *CXCL1*, **(G)** *ICAM-1*, and **(H)** *CXCL10* was quantified by RT-qPCR and normalized to *GAPDH*. Each dot represents one independent experiment. A p-value of <0.05 was considered statistically significant. P-values were determined by one-way ANOVA followed by Tukey’s *post hoc* test **(A, B, D, F-H)** or Kruskal– Wallis test followed by Dunn’s *post hoc* test **(C, E)**.

With studies reporting persistent circulation of spike protein in PCS patients, we examined the overall effect of recombinant S1 on the gene expression of markers indicating ED and immune activation in primary HRECs. Therefore, HRECs were mock-treated (negative control) or exposed to 100 ng/mL S1 or 100 ng/mL LPS (positive control) for 4 hours, and the mRNA expression of *VEGF*, *IL-6*, tumor necrosis factor (*TNF*), interleukin-8 (*IL-8*), *MCP-1*, C-X-C motif chemokine ligand 1 (*CXCL1*), *ICAM-1*, and *CXCL10* was assessed. While S1 induced substantial mRNA expression of *VEGF* (**Figure 3A**), a key regulator of angiogenesis, it did not significantly affect the expression of several inflammatory markers, including *IL-6*, *TNF*, *MCP-1*, *CXCL1*, *ICAM-1*, and *CXCL-10* (**Figures 3B, C, E-H**). Interestingly, *IL-8* expression significantly decreased after S1 exposure (**Figure 3D**).

### 3.4 S1 leads to HIF-1α and HIF-2α nuclear activation and downstream pathways rather than NF-κB in HRECs

**Figure 4:**
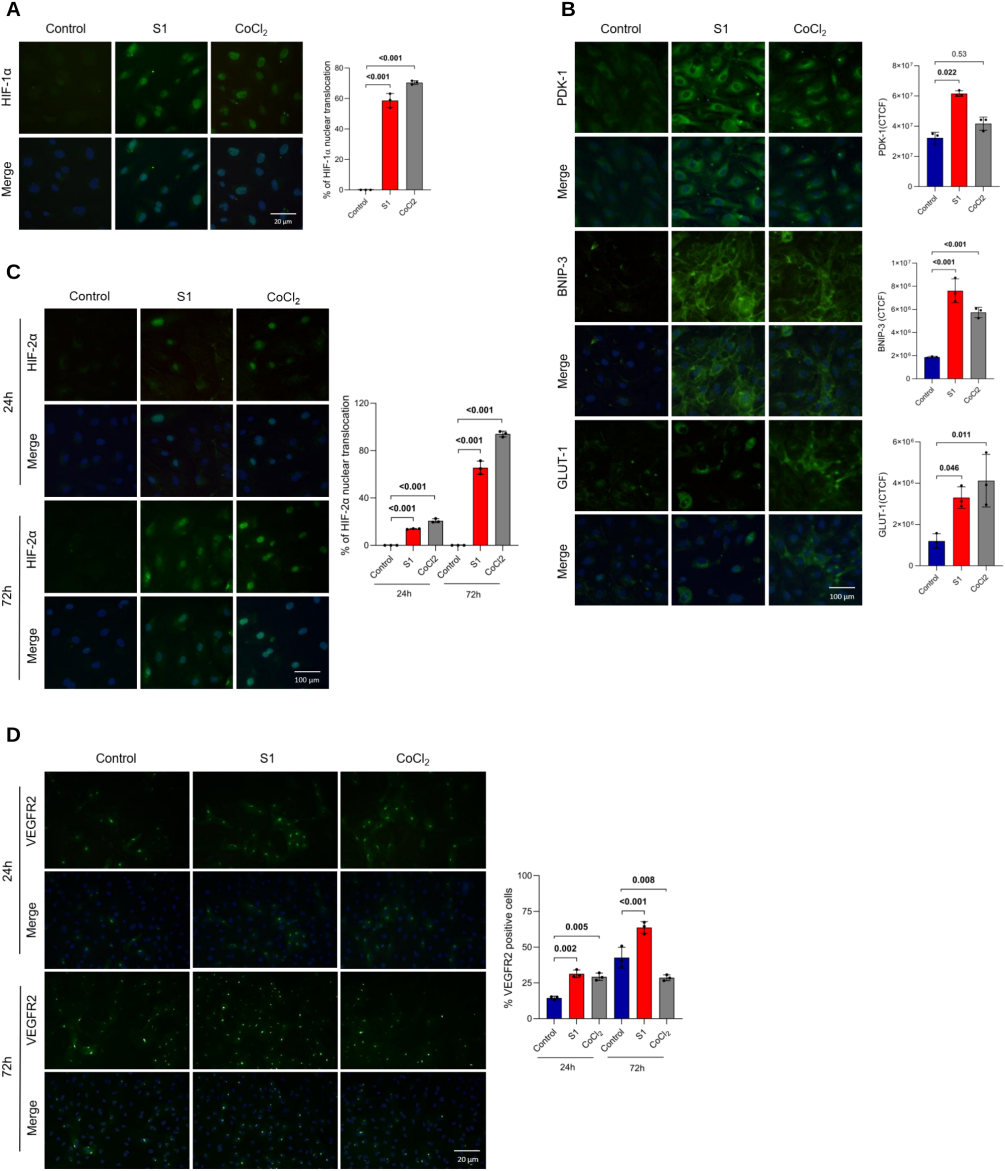
S1 induces high HIF-1/2α nuclear translocation and VEGFR2 upregulation in HRECs. HRECs were mock-treated (control), stimulated with S1 (100 ng/mL), or treated with CoCl_2_ (100 µM) for immunofluorescence analysis. **(A)** HIF-1α nuclear translocation after 8 h. **(B)** PDK-1, BNIP-3, and GLUT-1 expression after 24 h. **(C)** HIF-2α nuclear translocation after 24 h and 72 h. **(D)** VEGFR2 expression after 24 h or 72 h. All the primary antibodies were labelled with FITC (green), and the nuclei were counterstained with DAPI (blue). Images (left) were acquired at 20× magnification, and scale bars represent 20 µm **(A, D)** or 100 µm **(B, C)**. Graphs (right) illustrate the percentage of nuclear translocation **(A, C)**, the corrected total cell fluorescence **(B)**, and the percentage of positive cells **(D)**. Data are represented as means ± SD. Each dot represents one independent experiment. A p-value of <0.05 was considered statistically significant. P-values were determined by one-way ANOVA followed by Tukey’s *post hoc* test.

Given that S1 treatment induced substantial *VEGF* upregulation but failed to elicit the selected inflammatory markers described in **Figure 3**, we assessed the activation of HIF-1/2α and NF-κB in HRECs treated with S1. CoCl_2_, a well-known inhibitor of prolyl hydroxylase domain (PHD) enzymes that stabilizes HIF proteins and mimics hypoxic conditions (62), and LPS, a robust activator of the NF-κB signaling pathway (63), were used as positive controls. Nuclear expression of HIF-1α was absent in mock-treated cells. The cells treated with either recombinant S1 or CoCl2 exhibited significant activation of HIF-1α at 8 hours after treatment (**Figure 4A**).

To investigate the activation of HIF transcriptional pathways, we analyzed the expression of key HIF target proteins, namely phosphoinositide-dependent protein kinase 1 (PDK1), BCL2/adenovirus E1B 19 kDa protein-interacting protein 3 (BNIP3), and glucose transporter 1 (GLUT1). Our findings revealed that HRECs expressed high levels of the three selected markers under S1 exposure (**Figure 4B**).

S1-induced HIF-1α activation was completely absent at 24 hours post-treatment (**Supplementary Figure 2**). In contrast, HIF-2α was activated after 24 hours, an effect that was exacerbated after 72 hours (**Figure 4C**). In addition, S1 induced the expression of VEGFR2 at both the 24- and 72-hour time points (**Figure 4D**). These observations, coupled with the initial *VEGF* upregulation, suggest that S1 may promote angiogenesis by activating the HIF pathway and upregulating VEGFR2.

Notably, S1 did not induce NF-κB nuclear translocation response, whereas LPS induced a strong response at 4 hours post-treatment (**Supplementary Figure 3**). This result is consistent with our previous observation that the specific S1 concentration used in this study had a limited effect on the expression of the selected inflammatory markers.

### 3.5 S1 and plasma from PCS patients induce oxidative stress and are associated with alterations in endothelial barrier properties, an effect that is attenuated by pharmacologic inhibition of HIF-2α

**Figure 5:**
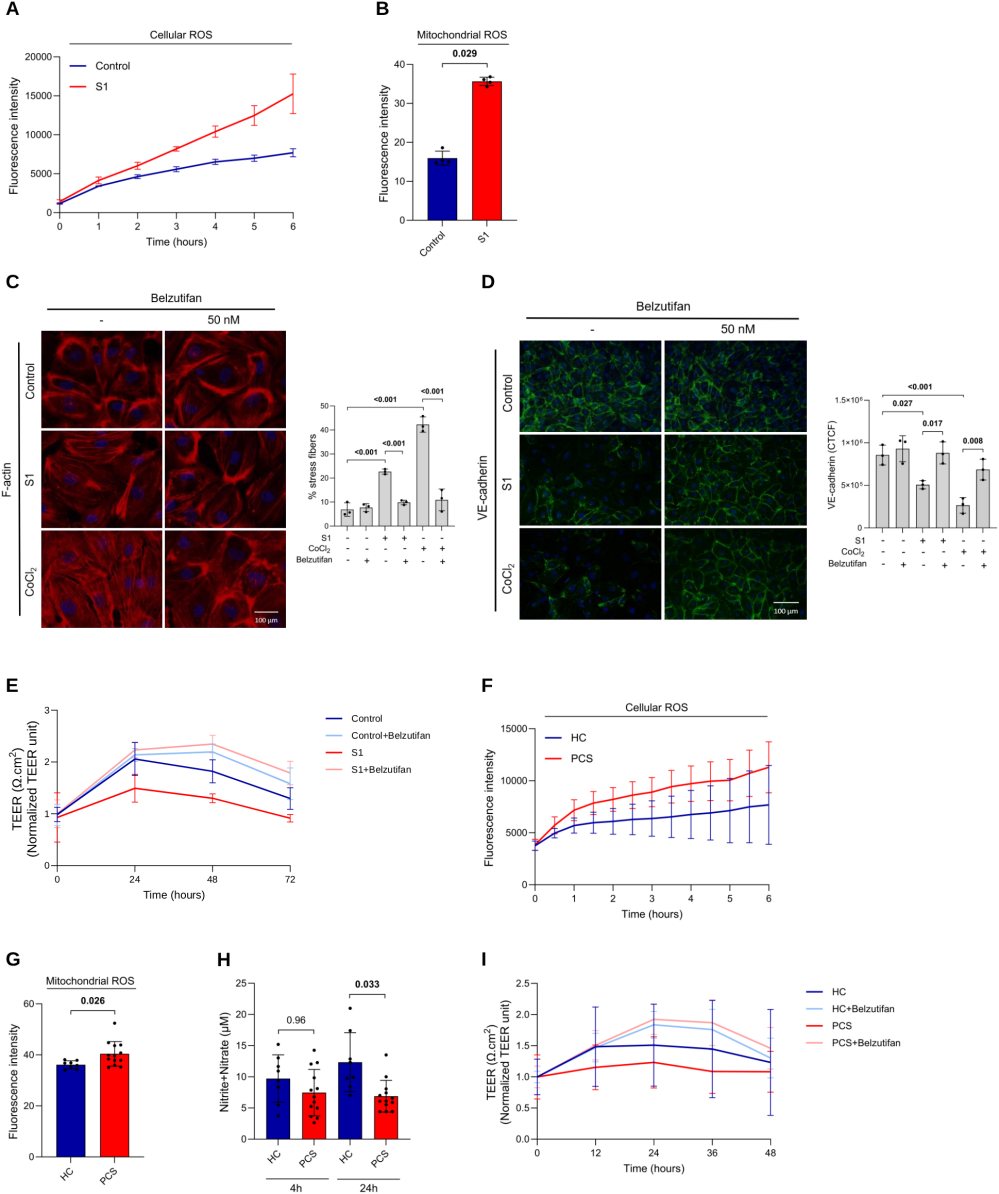
S1 and plasma from PCS patients influence ROS production, impair NO availability, and disrupt barrier integrity in HRECs, effects improved by belzutifan. **(A)** HRECs were mock-treated (control) or stimulated with S1 (100 ng/ml) for 0–6 h, and cellular ROS levels were measured using DCFDA/H_2_DCFDA (n = 3 independent experiments). **(B)** HRECs were mock-treated (control) or stimulated with S1 (100 ng/ml) for 4 h, and mitochondrial ROS production was measured by flow cytometric analysis using MitoSox Red (n = 4 independent experiments). **(C, D)** HRECs were mock-treated (control), stimulated with S1 (100 ng/mL) or CoCl_2_ (100 µM), and treated with belzutifan (50 nM) for 72 h. Immunofluorescence staining was performed for F-actin **(C, **red**)** and VE-cadherin **(D, **green**)**, with nuclei counterstained with DAPI (blue). Images **(left)** were acquired at 20× magnification, and scale bars represent 100 µm. Graphs (right) illustrate the percentage of positive cells **(C)** and the corrected total cell fluorescence (CTCF) **(D)** (n = 3 independent experiments). **(E)** HRECs were cultured at confluence on ECIS electrodes and then stimulated with 100 ng/mL S1 or left untreated in the presence or absence of 50 nM belzutifan for 0–72 h. The loss of barrier integrity was determined by transendothelial electrical resistance (TEER). Values were normalized to time = 0 for easier comparisons (n = 3 independent experiments). **(F)** HRECs were treated with 2% plasma from healthy individuals (HC, n=8) or PCS patients (n=13) for 0–6 h, and cellular ROS levels were measured using DCFDA/H_2_DCFDA. **(G)** Mitochondrial ROS production in HRECs exposed to 2% plasma from HC (n=8) or PCS patients (n=13) for 4 h, measured by flow cytometric analysis using MitoSox Red. **(H)** Total NO levels in HRECs exposed to 2% plasma from HC (n=8) or PCS patients (n=13) for 4 h and 24 h, measured using a fluorometric assay for total nitrite/nitrate levels. **(I)** HRECs were cultured at confluence on ECIS electrodes and exposed to 2% plasma from HC or PCS patients in the presence or absence of 50 nM belzutifan for 0–48 h. The loss of barrier integrity was determined by transendothelial electrical resistance (TEER). Values were normalized to time = 0 for easier comparisons. Data are represented as means ± SD. Each dot represents one independent experiment for S1 studies or one individual donor for plasma studies. A p-value of <0.05 was considered statistically significant. P-values were determined by two-way ANOVA followed by Tukey’s *post hoc* test **(A, E, F, I)**, Mann–Whitney U test **(B)**, one-way ANOVA followed by Tukey’s *post hoc* test **(C, D)**, Student’s t-test **(G)**, and Kruskal–Wallis test followed by Dunn’s *post hoc* test **(H)**. .

Next, we tested whether S1 exposure affects ROS and NO production in HRECs. ROS significantly increased in a time-dependent manner (3 hours, p=0.014; 4 hours, p= 0.008; 5 hours, p=0.010; and 6 hours, p=0.025) upon S1 treatment (**Figure 5A**). Additionally, mitochondrial ROS production also increased in cells treated with S1 (**Figure 5B**). S1 reduced NO production; however, this effect was not significant (**Supplementary Figure 4**).

Following our observations, we investigated whether S1 alone is capable of altering endothelial barrier integrity and function. HRECs were treated with or without S1 for 72 hours before immunofluorescence staining was performed to detect F-actin formation and VE-cadherin and claudin-5 distribution in HRECs. F-actin in the control condition was localized cortically (**Figure 5C**). In contrast, exposure to S1 diminished cortical actin and resulted in an elongated and narrower cell morphology (**Figure 5C**). S1 treatment triggered cytoskeletal remodeling, characterized by the formation of prominent actin stress fibers (**Figure 5C**). Furthermore, mock-treated cells displayed almost intact VE-cadherin (**Figure 5D**) and claudin-5 (**Supplementary Figure 5**) bordering cells in unbroken, confluent monolayers. Upon treatment with S1, there was a significant reduction in VE-cadherin and claudin-5, with gaps appearing between cells (**Figure 5D**; **Supplementary Figure 5**). In line with these findings, TEER of the cell monolayer significantly decreased in S1-treated cells (**Figure 5E**; **Table 1**), confirming the loss of endothelial barrier integrity.

**Table 1:** TEER effect sizes and pairwise comparisons in HRECs exposed to S1.

Condition/comparison		T:0	T:24h	T:48h	T:72h
Panel A. Effect sizes (% changed at predefined timepoint and AUC interval)					
Control	%Δ at 48 h *vs* T0	—	—	+82.1%	—
Control+Belzutifan	%Δ at 48 h *vs* T0	—	—	+119.6%	—
S1	%Δ at 48 h *vs* T0	—	—	+30%	—
S1+Belzutifan	%Δ at 48 h *vs* T0	—	—	+134.7%	—
Control	AUC0–72h (95% CI)	120.5 (106.3–134.7)			
Control+Belzutifan		134.7 (123.9–145.6)			
S1		89.2 (74.56–103.9)			
S1+Belzutifan		143.4 (132.2–154.6)			
Panel B. Adjusted p-values (Condition/comparison)					
Control *vs* Control+Belzutifan	Adjusted p	>0.99	0.98	0.22	0.45
Control *vs* S1		0.99	**0.022**	**0.048**	0.21
S1 *vs* Control+Belzutifan		0.99	**0.010**	**<0.001**	**0.007**
S1 *vs* S1+Belzutifan		0.98	**0.003**	**<0.001**	**<0.001**

Summary of the normalized TEER: TEER was normalized to baseline (T0 = 1). Panel A shows quantitative effect sizes: percentage change from baseline at the predefined time point (48 h; calculated as (nTEER48h−1)×100) and area under the TEER–time curve from 0–72 h (AUC0–72h) with 95% confidence intervals. Panel B shows Tukey-adjusted p-values from two-way ANOVA for the indicated pairwise comparisons at each time point. A p-value of <0.05 was considered statistically significant. TEER experiments were performed in three independent ECIS runs; within each run, each condition was measured in multiple wells. Experimental conditions are as in the corresponding TEER figure legend.Bold values indicate statistically significant results.

Next, we hypothesized that by inhibiting HIF-2α, we could partially reverse the damage inflicted by S1 on the monolayer barrier. To test this hypothesis, we treated HRECs exposed to S1 or CoCl2 with belzutifan for 72 hours and assessed HIF-2α activation, stress fiber production, and VE-cadherin and claudin-5 expression. We observed that the HIF-2α activation induced by either S1 or CoCl2 was completely inhibited by belzutifan (**Supplementary Figure 6**). Moreover, we observed that the excessive generation of stress fibers in cells treated with S1 or CoCl2 was significantly reduced (**Figure 5C**). In addition, the decrease in VE-cadherin expression was reversed, with VE-cadherin levels returning nearly to those of the control (**Figure 5D**). Inhibition of HIF-2α pathway with Belzutifan reversed the decrease in claudin-5 expression caused by S1 to levels similar to those in the control (**Supplementary Figure 5**). The endothelial integrity of HRECs was markedly greater in cells treated with belzutifan, as evidenced by TEER (**Figure 5E**; **Table 1**). Effect size analyses (percentage change at 48 h and AUC0–72h) further illustrate the magnitude and persistence of S1-induced barrier impairment and its reversal by belzutifan (**Table 1**). The integrity of HRECs exposed to S1 or belzutifan was not affected by changes in cell survival, as neither S1 nor belzutifan was cytotoxic (**Supplementary Figure 7A**) or influenced endothelial viability (**Supplementary Figure 7B**). These findings suggest that HIF-2α is involved in S1-induced endothelial barrier impairment and dysfunction and that inhibition of HIF-2α signaling improves monolayer integrity.

Owing to the technical challenges of investigating ED at the cellular level *in vivo*, we used an *in vitro* approach in which HRECs were exposed to 2% plasma from patients with PCS or HCs to assess correlations between ED, increased ROS production, and impaired NO synthesis. Basic characteristics were not significantly different between the two cohorts (**Supplementary Table 4**). As we observed associations between PCS severity and ED, plasma samples from PCS patients were selected solely on the basis of their severity score. Our findings revealed a time-dependent increase in cellular ROS (1 hour, p=0.0086; 2 hours, p=0.0064; 3 hours, p=0.0.0083; 4 hours, p=0.012; 5 hours, p=0.0321; and 6 hours, p=0.0298) following treatment with PCS-plasma (**Figure 5F**). Additionally, mitochondrial ROS levels significantly increased at 4 hours post-treatment (**Figure 5G**). Consequently, NO production decreased in HRECs exposed to PCS-plasma for 4 hours and 24 hours, but reached significance only after 24 hours of exposure compared with that in HC-treated cells (**Figure 5H**). The delayed decrease in NO levels may reflect a self-amplifying mechanism described in prior literature, in which early ROS generation can promote progressive eNOS uncoupling, further amplifying oxidative stress and reducing NO bioavailability over time (64, 65); however, this mechanistic interpretation was not directly tested in the present study.

To investigate whether plasma from PCS patients compromises endothelial barrier function, we measured TEER. We observed a non-significant TEER reduction with PCS-plasma in a direction similar to S1. Moreover, belzutifan significantly improved barrier integrity relative to PCS baseline, as shown by the significant TEER increase (**Figure 5I**; **Table 2**). Corresponding effect size measures (percentage change at 24 h and AUC0–48h) support a modest attenuation of barrier function by PCS plasma and a significant functional improvement with HIF-2α inhibition (**Table 2**). Exploratory correlation analyses were performed to examine associations between plasma biomarkers and plasma-induced endothelial functional readouts in PCS patients. Plasma VEGF levels were positively correlated with TEER slope, while plasma VCAM-1 levels were inversely correlated with endothelial NO production. A positive association between VEGF and NO production was also observed. No significant correlations were detected between other plasma biomarkers and endothelial oxidative stress or barrier function measures (**Supplementary Figure 8**).

**Table 2:** TEER effect sizes and pairwise comparisons in HRECs exposed to plasma.

Condition/comparison	Adjusted p	T:0	T:12h	T:24h	T:36h	T:48h
Panel A. Effect sizes (% changed at predefined timepoint and AUC interval)						
HC (n=8)	%Δ at 24 h *vs* T0	—	—	+51%	—	—
HC+Belzutifan (n=6)	%Δ at 24 h *vs* T0	—	—	+83.6%	—	—
PCS (n=10)	%Δ at 24 h *vs* T0	—	—	+23,2%	—	—
PCS+Belzutifan (n=9)	%Δ at 24 h *vs* T0	—	—	+92.5%	—	—
HC	AUC0–48h (95% CI)	66.68 (44.04–89.32)				
HC+Belzutifan		74.61 (66.12–83.10)				
PCS		54.14 (41.93–66.35)				
PCS+Belzutifan		78.38 (68.99–87.78)				
Panel B. Adjusted p-values						
HC *vs* HC+Belzutifan	Adjusted p	>0.99	0.99	0.32	0.51	0.99
HC *vs* PCS		>0.99	0.65	0.74	0.59	0.97
PCS *vs* HC+Belzutifan		>0.99	0.37	0.11	0.12	0.49
PCS *vs* PCS+Belzutifan		>0.99	**0.007**	**0.003**	**0.008**	0.13

Summary of the normalized TEER: TEER was normalized to baseline (T0 = 1). Panel A shows quantitative effect sizes: percentage change from baseline at the predefined time point (24 h; calculated as (nTEER24h−1)×100) and area under the TEER–time curve from 0–48 h (AUC0–48h) with 95% confidence intervals. Panel B shows Tukey-adjusted p-values from two-way ANOVA for the indicated pairwise comparisons at each time point. A p-value of <0.05 was considered statistically significant. TEER experiments were performed across three independent ECIS runs due to plate capacity constraints. Each plasma sample was applied to one well per condition. n refers to the number of independent plasma samples in each group. Experimental conditions are as in the corresponding TEER figure legend.Bold values indicate statistically significant results.

### 3.6 Plasma-induced endothelial oxidative stress correlates with clinical disease severity

To explore whether plasma-induced ED reflects clinical disease burden, we performed exploratory Spearman correlation analyses between disease severity scores and endothelial functional readouts in the combined cohort (PCS and HCs). Higher PCS severity scores were associated with increased cellular ROS and mitochondrial ROS production, while C19-YRS scores similarly correlated with cellular ROS generation. In contrast, no significant associations were observed between severity scores and NO production or endothelial barrier function metrics, including TEER dynamics (**Table 3**).

**Table 3:** Exploratory correlations between disease severity scores and plasma-induced endothelial functional readout.

Severity_score	Endpoint	n	ρ	p
**PCS severity score**	**Cellular ROS (4 h)**	**21**	**0.57**	**0.007**
**PCS severity score**	**Mitochondrial ROS (4 h)**	**21**	**0.51**	**0.018**
PCS severity score	NO (4 h)	21	-0.23	0.320
PCS severity score	ΔTEER (0–12 h)	18	-0.13	0.595
PCS severity score	ΔTEER (0–24 h)	18	-0.11	0.663
PCS severity score	TEER slope	18	0.03	0.891
**C19-YRS**	**Cellular ROS (4 h)**	**21**	**0.52**	**0.015**
C19-YRS	Mitochondrial ROS (4 h)	21	0.35	0.123
C19-YRS	ΔTEER (0–12 h)	18	-0.29	0.248
C19-YRS	ΔTEER (0–24 h)	18	-0.27	0.277
C19-YRS	NO (4 h)	21	-0.25	0.284
C19-YRS	TEER slope	18	-0.13	0.593

Spearman correlation analyses between clinical disease severity scores: The PCS severity score and COVID-19 Yorkshire Rehabilitation Scale [C19-YRS] and plasma-induced endothelial functional readouts, including cellular and mitochondrial ROS, NO production, and TEER dynamics. Unadjusted Spearman correlation coefficients (ρ), sample sizes (n), and p-values are shown. Analyses were performed using pairwise complete observations and are intended as hypothesis-generating.Bold values indicate statistically significant results.

## 4 Discussion

As evidence arises that persistent viral SARS-CoV-2 RNA and proteins have been reported in some individuals following acute infection (10, 66), the primary aim of this work was to examine the effect of the S1 antigen on endothelial function using primary HRECs *in vitro*. In this study we demonstrated that S1-driven activation of the HIF pathways leads to ED, which is characterized by increased oxidative stress and barrier dysfunction. Pharmacologic inhibition of HIF-2α with belzutifan modulated endothelial barrier integrity in this experimental setting.

In our PCS cohort, VEGF and MCP-1 levels were elevated, which is consistent with reports of higher ED markers in PCS (67–69). Given their roles in endothelial activation and vascular remodeling, persistent elevation of VEGF and MCP−1 is consistent with ongoing ED. Studies have reported that PCS patients frequently exhibit higher anti-S1 IgG antibody levels and/or viral coinfections, which are associated with chronic inflammation, autoimmunity, and sustained endothelial activation (70–72). In exploratory analyses, anti-S1 IgG levels were positively correlated with VEGF in PCS patients, indicating a potential association between spike-directed humoral responses and VEGF signaling. Vaccination status and number of prior infections varied within the PCS cohort. Although anti-S1 IgG levels did not differ across these factors, residual confounding related to cumulative antigen exposure cannot be fully excluded.

Endotheliitis in acute SARS-CoV-2 infection contributes to vascular complications. Unexpectedly, S1 alone did not induce the mRNA expression of the selected inflammatory markers or NF-κB activation in HRECs. These findings suggest that inflammatory pathway activation in HRECs may require higher S1 concentrations and/or occur indirectly via immune cell activation. On the other hand, S1 treatment induced *VEGF* mRNA expression and VEGFR2 activation. Moreover, S1-induced HIF-1α nuclear translocation peaked at 8 hours but was completely absent at 24 hours, whereas HIF-2α activity persisted up to 72 hours, suggesting a shift from HIF-1α to HIF-2α prolonged response (38, 39, 73). This temporal pattern is in line with the HIF switch described in ECs under prolonged hypoxia, where HIF-1α mediates acute responses within 24 hours, and HIF-2α drives sustained vascular adaptations beyond 24 hours, including VEGF/VEGFR signaling (39, 74). These observations suggest that *VEGF* expression and VEGFR2 activation are driven by S1-induced activation of HIF-1α and HIF-2α rather than the NF-κB pathway. In support of these findings, we observed a downregulation of *IL-8* mRNA expression by S1, possibly due to HIF-1α activation, as HIF-1α is known to inhibit NF-E2-related factor, a transcription factor that modulates *IL-8* expression in ECs (75, 76). Additionally, a recent analysis of the plasma proteome of PCS patients revealed enrichment of HIF signaling pathways (37). The prolonged activation of both HIF-2α and VEGFR2 observed following S1 exposure may lead to abnormal angiogenesis, oxidative stress, secondary inflammatory signaling, and thromboinflammation, mechanisms that have been previously described in PCS, highlighting ED as a central driver of PCS symptoms (21).

S1 has been hypothesized to interact with Hb, disrupting its function, reducing oxygen transport, and inducing hypoxia (77, 78). Since hypoxia is a known driver of oxidative stress, reduced NO bioavailability, and activation of HIFs, it can consequently impair endothelial barrier function, disrupt vascular homeostasis, and contribute to ED (79). In exploratory analysis we found a weak albeit significant association between higher anti-S1 IgG levels and low Hb in PCS patients. Hb and Hct were negatively correlated with PCS severity, which suggests a potential role of chronic hypoxia in the development of PCS symptoms. We observed significantly higher EPO levels in PCS patients, which is consistent with altered oxygen-sensing and erythropoietic regulation. This aligns with a study reporting persistent iron dysregulation and inflammatory anemia associated with PCS and studies reporting higher EPO levels in PCS patients (68, 80).

Recent studies have suggested that S1 can influence blood–brain barrier (BBB) integrity and that persistent spike protein exposure may be associated with cognitive symptoms in PCS (81, 82). Given the microvascular involvement and neurological manifestations observed in PCS, HRECs were selected as our *in vitro* model due to the accessibility and well-defined organization of the retina, which provides a convenient model of microvascular endothelium. This choice was further motivated by our prior clinical work demonstrating persistent retinal microvascular dysfunction in PCS, as assessed by retinal vessel analysis (20). In addition, retinal endothelial cells share several morphologic and functional features with brain microvascular endothelial cells (83–85). However, they remain a specialized vascular bed and do not fully recapitulate endothelial properties of other vascular territories. Our experiments demonstrated that S1 induces cellular and mitochondrial ROS in HRECs, a critical mechanism underlying endothelial damage, as ROS can disrupt the cytoskeleton, degrade tight junction proteins, and impair barrier integrity (86). S1-treated HRECs exhibited an elongated morphology with loss of cortical actin rim and formation of prominent stress fibers, consistent with ROS-mediated endothelial injury. This was accompanied by diminished VE-cadherin and claudin-5 at cell-cell contacts and a significant decrease in TEER. TEER reflects the ability of ECs to form inter-endothelial junctions and maintain a selective barrier between the bloodstream and surrounding tissues. A decrease in TEER, associated with the formation of stress fibers and the decrease of endothelial cell junction proteins, confirms that S1 induces monolayer permeability and compromises barrier function, hallmarks of ED (87, 88). The improvement of TEER after belzutifan treatment is likely mediated through mechanisms such as inhibiting HIFs, reducing oxidative stress, and stabilizing cell-cell junctions. These findings provide insight into mechanisms by which S1-induced endothelial stress may impair barrier integrity *in vitro* and suggest potential relevance to BBB-associated processes observed in PCS (89, 90), although this was not directly assessed here.

Similarly, we showed that HRECs exposed to plasma from PCS patients exhibited a notable increase in cellular and mitochondrial ROS production and a pronounced reduction in NO synthesis. TEER from monolayers exposed to PCS-plasma showed visible but non-significant endothelial barrier disruption compared with that from monolayers exposed to HC-plasma. The alignment of these findings with those observed in S1-treated HRECs suggests that S1 may contribute to endothelial alterations observed with PCS plasma. Exploratory correlations indicated that higher plasma VEGF levels were associated with increased NO bioavailability and a more positive TEER slope in endothelial monolayers. These associations are compatible with VEGF–eNOS crosstalk and suggest that, in this experimental context, VEGF signaling may reflect adaptive or reparative endothelial responses rather than acute permeability induction. Depending on signaling balance and oxidative milieu, VEGF can promote endothelial remodeling and junctional reorganization, which may explain its association with improved TEER dynamics and NO production observed here. Additionally, higher PCS severity (C19YRS and PCS severity score) in patients was associated with greater plasma-induced cellular and mitochondrial ROS generation in ECs, consistent with prior clinical studies linking PCS symptom burden and impaired recovery to increased oxidative/nitro-oxidative stress and reduced antioxidant defenses (91–93). Because plasma samples used for these assays were derived from patients with higher PCS severity, the observed endothelial responses likely reflect the integrated effects of multiple circulating mediators, including inflammatory cytokines, autoantibodies, and potentially residual viral components, rather than S1 alone. Accordingly, these findings should be interpreted as reflecting endothelial responses associated with more severe PCS, while the relative contribution of individual plasma components to the endothelial phenotype warrants further investigation.

We propose that microvascular impairment in PCS might be a consequence of persisting viral components, such as S1, resulting in ongoing chronic inflammation, inflammatory anemia, and potential complement-mediated tissue injury (70, 71, 94). These mechanisms are not SARS-CoV-2 specific and have been observed in other chronic viral infections, such as CMV, EBV, and HHV-6 (95, 96). The overlap between PCS and myalgic encephalomyelitis/chronic fatigue syndrome (ME/CFS) is also well described, with similar findings of decreased NO production in ECs exposed to ME/CFS patient plasma and associations between the activation of HIF-2α and ME/CSF (55, 80, 97, 98).

The strengths of our study include the well-characterized PCS cohort with patient-reported outcome measures and comprehensive *in vitro* mechanistic studies of S1-induced HIF-2α signaling and endothelial barrier dysfunction in HRECs. Nevertheless, this study has some limitations. Direct S1/spike quantification in plasma was not performed due to laboratory resource limitations. Therefore, its presence, concentration, and functional contribution in our cohort cannot be confirmed, and future studies should incorporate such measurements. *In vitro* experiments used plasma from high-severity PCS patients only, limiting generalizability to milder cases. Additionally, we lack data from individuals who have recovered from COVID-19 or have been vaccinated, which could provide further context on the role of persistent viral components in PCS. All functional experiments in this study were conducted in primary HRECs, a specialized microvascular bed that may not fully recapitulate endothelial behavior in pulmonary, cardiac, renal, or cerebral vasculature. No experiments were performed in brain ECs, astrocytes, pericytes, or dedicated *in vitro* BBB models; therefore, any implications for BBB integrity or cognitive impairment are hypothesis-generating. Furthermore, there are inherent differences between *in vitro* models and human physiology, including the lack of systemic factors and immune responses in our experimental setup. Another limitation was the inability to measure VEGF at the protein level, as concentrations in both the control and S1-treated conditions were below the detection limit of the ELISA kit. We also acknowledge that the S1 concentration and exposure durations used in our experiments may not fully replicate the conditions seen in patients with PCS. Moreover, we did not include an irrelevant recombinant protein control or directly test endotoxin in the S1 preparation; while NF-κB/LPS controls argue against endotoxin as the primary driver, contaminant effects cannot be fully excluded. Finally, HIF−2α dependence is inferred from pharmacological sensitivity to belzutifan and should ideally be confirmed using genetic approaches in future work. HIF-2α inhibition improved barrier function *in vitro*, but lacks *in vivo* validation. Moreover, chronic HIF−2α inhibition may carry risks, including anemia and hypoxia (99), that would require careful benefit–risk evaluation. Preclinical animal studies and clinical trials are needed to establish the safety and efficacy of HIF-2α inhibition in PCS.

Taken together, these findings suggest that SARS-CoV-2 spike S1 activates a HIF-2α-dependent pathway in retinal endothelial cells that may contribute to the ED observed in PCS. S1 induces ROS production and HIF-1α/HIF-2α signaling, leading to VEGFR2 upregulation, cytoskeletal remodeling, and barrier disruption, effects ameliorated by HIF-2α inhibition. These *in vitro* observations provide a mechanistic framework for investigating microvascular endotheliopathy in PCS and support future studies to further define the relevance of this pathway to PCS-associated ED.

## Data Availability

The raw data supporting the conclusions of this article will be made available by the authors, without undue reservation.
