# Supplementary material for: SARS−CoV−2 spike S1-mediated HIF−2α activation in retinal endothelial cells suggests a mechanism contributing to post−COVID endothelial dysfunction
Source: Front Immunol. 2026 Mar 9;17:1770758. doi: 10.3389/fimmu.2026.1770758 (PMC13006635; doi:10.3389/fimmu.2026.1770758)
Supplement: Supplementary file 1 [file Table1.docx]

Supplementary Material

**Supplementary Methods**

**Cytotoxic assay:** 20,000 cells were seeded in a 96 well plate, in 100 µl of medium without phenol red and allowed to adhere overnight. HREC were mock-treated, or exposed to 100 ng/mL of S1 in the presence or absence of 50 nm Belzutifan for 72 hours. After the incubation time, 50 µl of the medium was collected from the sample plate and transferred to a new clear 96 well plate. The Cytotoxicity Detection Kit from Roche (LDH assay) was used following the manufacturer’s instructions. Absorbance was measured at 490 nm, every 5 minutes for a period of 30 minutes in an Infinite M Plex microplate reader (Tecan). Triton X-100 was used as positive control. To calculate the percentage of toxicity after LDH assay, the following formula was used: $Toxicity \left( \% \right)=\frac{Experimental absorbance-Untreated cells absorbance}{Triton X absorbance-Untreated cells absorbance}\times100$.

**Viability assay:** 20,000 cells were seeded in a 96 well plate, in 100 µl of medium without phenol red and allowed to adhere overnight. The next day, HREC were mock-treated, or exposed to 100 ng/mL of S1 in the presence or absence of 50 nm Belzutifan for 72 hours. After incubation period, 15µl of the diluted tetrazolium salt (3-(4,5-dimethylthiazol-2-yl)-2,5-diphenyltetrazolium bromide powder (MTT) (Sigma) was added to each well and kept at 37 °C for 3 hours. At the end of the 3 hours incubation, 100 µl of stop solution composed by 20% of Triton X, 20 % of HCL and 60 % of Isopropanol was added. Plate was kept at 37 °C, protected from light, for 4 hours. The absorbance was measured at 570 nm with a reference wavelength of 650 nm in an Infinite M Plex microplate reader (Tecan). Triton X was used as negative control. The percentage of cell viability was measured using the following formula: $Cell viability \left( \% \right)=\frac{Absorbance of treated cells}{Absorbance of untreated cells}\times100$.

**Supplementary Figures**

**Supplementary Figure 1**

**Supplementary Figure 1. Correlations between S1-specific humoral response or PCS severity and anemia and VEGF.** Scatterplots show spearman (A-C) and pearson (D-E) correlations between VEGF (n=41), Haemoglobin (Hb, n=37), Haematocrit (Hct, n=37) and Anti-S1 IgG (n=41) or COVID-19 Yorkshire Rehabilitation Scale (C19-YRS, n=40) and the corresponding R and p-value. The black line represents the linear regression line with the grey shaded area indicating the 95% confidence interval.

**Supplementary Figure 2**


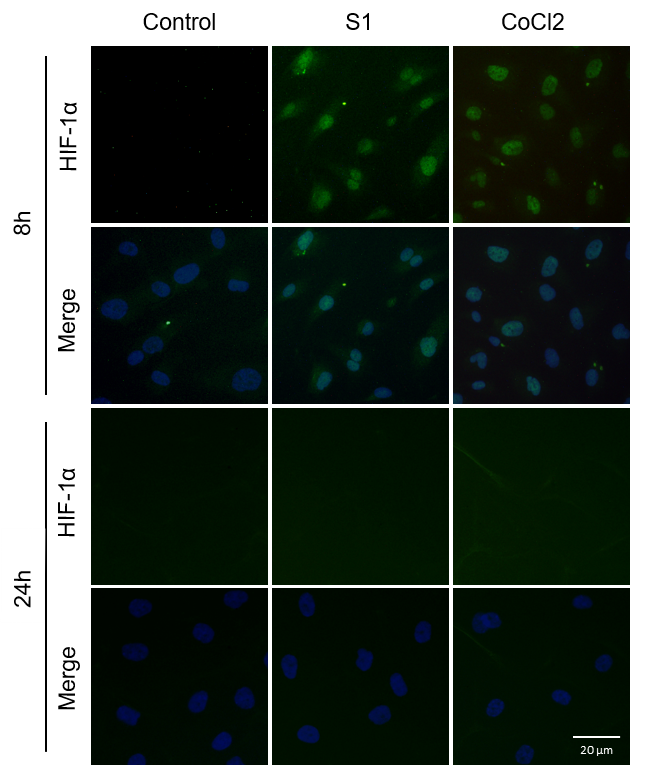


**Supplementary Figure 2. Maximum activation of HIF-1α at 8 hours and absence at 24 hours.** Immunofluorescence staining of HRECs, mock-treated (control) or exposed to S1 at 100 ng/mL or CoCl_2_ at 100 µM, shows a strong activation of HIF-1α (FITC, green) at 8 hours post-treatment with S1 and CoCl_2_. On the contrary, activation is absent after 24 hours. The nuclei were counterstained with DAPI (blue). Images were acquired at 20× magnification and scale bar represents 20 µm.

**Supplementary Figure 3**

**
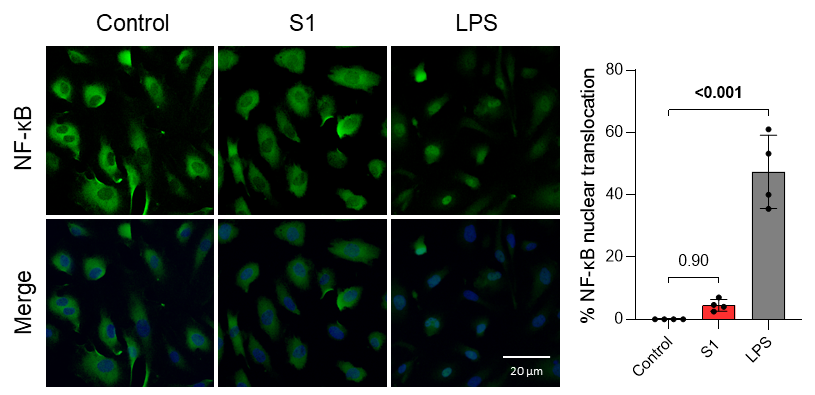
Supplementary Figure 3. HRECs treated with S1 show a markedly less pronounced NF-κB nuclear translocation when compared with cells treated with LPS.** Representative confocal analysis of NF-κB (FITC, green) in HRECs, mock-treated (control, n=4), or exposed to 100 ng/mL S1 (n=4) or 100 ng/mL LPS (n=4) for 4 hours. The nuclei were counterstained with DAPI (blue). Images (left) were acquired at 20× magnification and scale bar represents 20 µm. Graph (right) illustrates the percentage of nuclear translocation. Data are shown as means ± SD. A p-value of <0.05 was considered statistically significant. P-values were determined by 1-way ANOVA followed by Tukey’s post hoc test

**Supplementary Figure 4**

**
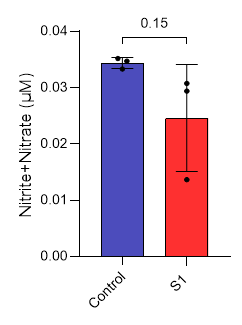
**

**Supplementary Figure 4. S1 induces a noticeable albeit not significant reduction in NO production in HRECs.** Total NO level measurement of HRECs, mock-treated (Control, n=3) or exposed to S1 at 100 µg/mL (n=3) for 4 hours, using a fluorometric-based assay that measures total nitrite/nitrate levels. Data are shown as means ± SD. A p-value of <0.05 was considered statistically significant. P-value was determined by Student’s t- test

**Supplementary Figure 5**


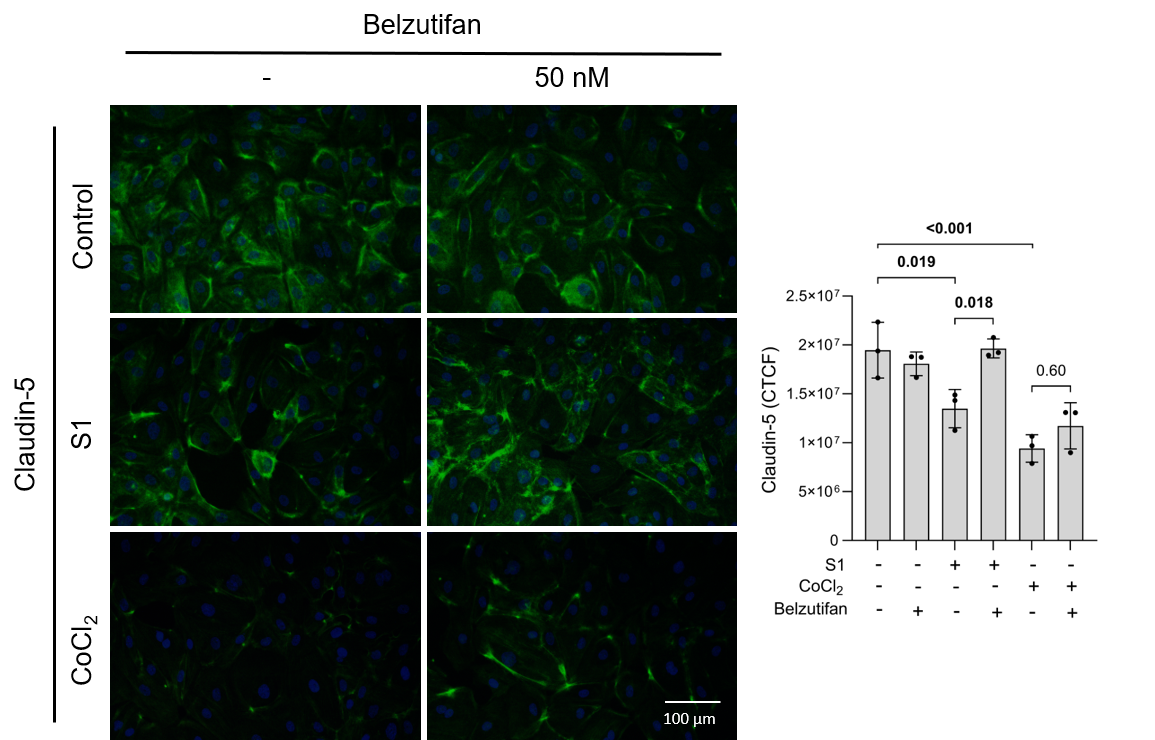


**Supplementary Figure 5. HRECs treated with S1 show a decreased expression of Claudin-5, an effect that is restored by Belzutifan.** Immunofluorescence staining for Claudin-5 (FITC, green) in HRECs, mock-treated (Control, n=3), or exposed to 100 ng/mL S1 (n=3) or 100 µM CoCl_2_ (n=3), and treated with 50 nM Belzutifan (n=3) for 72 hours. Nuclei were labelled with DAPI (blue). Images were acquired at 20× magnification and scale bar represents 100 µm. Graph (right) illustrates the corrected total cell fluorescence. Data are shown as means ± SD. A p-value of <0.05 was considered statistically significant. P-values were determined by 1-way ANOVA followed by Tukey’s post hoc test

**Supplementary Figure 6**


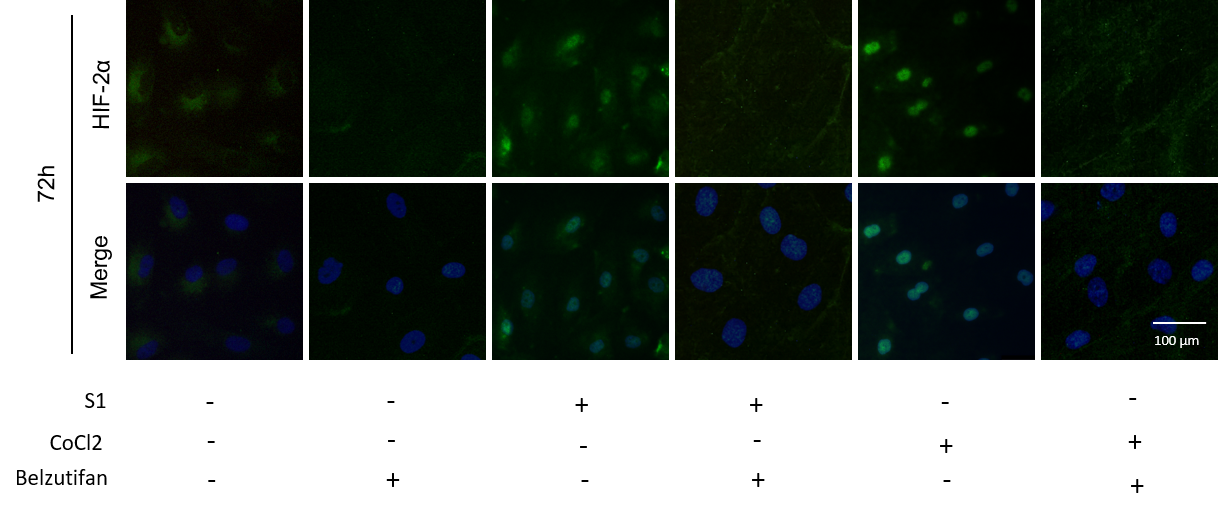


**Supplementary Figure 6. Belzutifan totally inhibits HIF-2α activation**. HRECs, mock-treated (Control, n=8), or exposed to 100 ng/mL S1 (n=8) or 100 µM CoCl_2_ (n=8), and treated with 50 nM Belzutifan (n=8) for 72 hours show HIF-2α (FITC, green) nuclear translocation upon S1 and CoCl_2_ stimulation that is completely inhibited by the treatment with 50 nM Belzutifan. Nuclei were labelled with DAPI (blue). Images were acquired at 20× magnification and scale bar represents 100 µm

**Supplementary Figure 7**

**
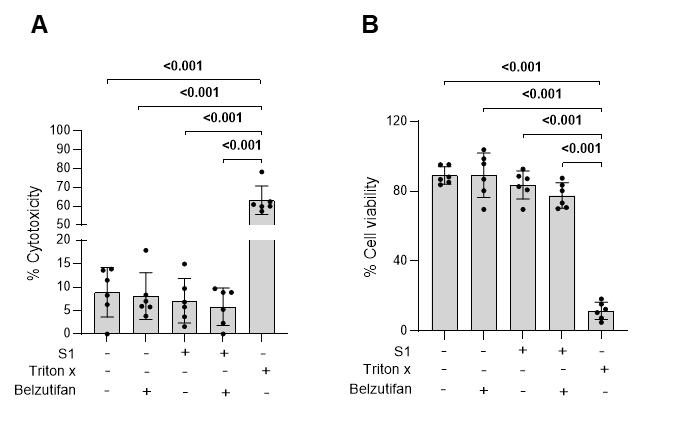
**

**Supplementary Figure 7. Neither S1 nor Belzutifan is cytotoxic to HRECs or changes cells viability.** HRECs were stimulated with or without 100 ng/ mL S1 in the presence or absence of 50 nM Belzutifan, for 72 hours. n=6 biological replicates were used for each group (A and B). Cytotoxicity assay (A) and viability assay (B) showed that there were no significant differences between control, S1, Belzutifan and S1 with Belzutifan. Triton X was used as a positive control and showed significant cytotoxicity (A) and significantly less cell viability (B) compared to all other groups. Data are shown as means ± SD. A p-value of <0.05 was considered statistically significant. P-values were determined by 1-way ANOVA followed by Tukey’s post hoc test

**Supplementary Figure 8**


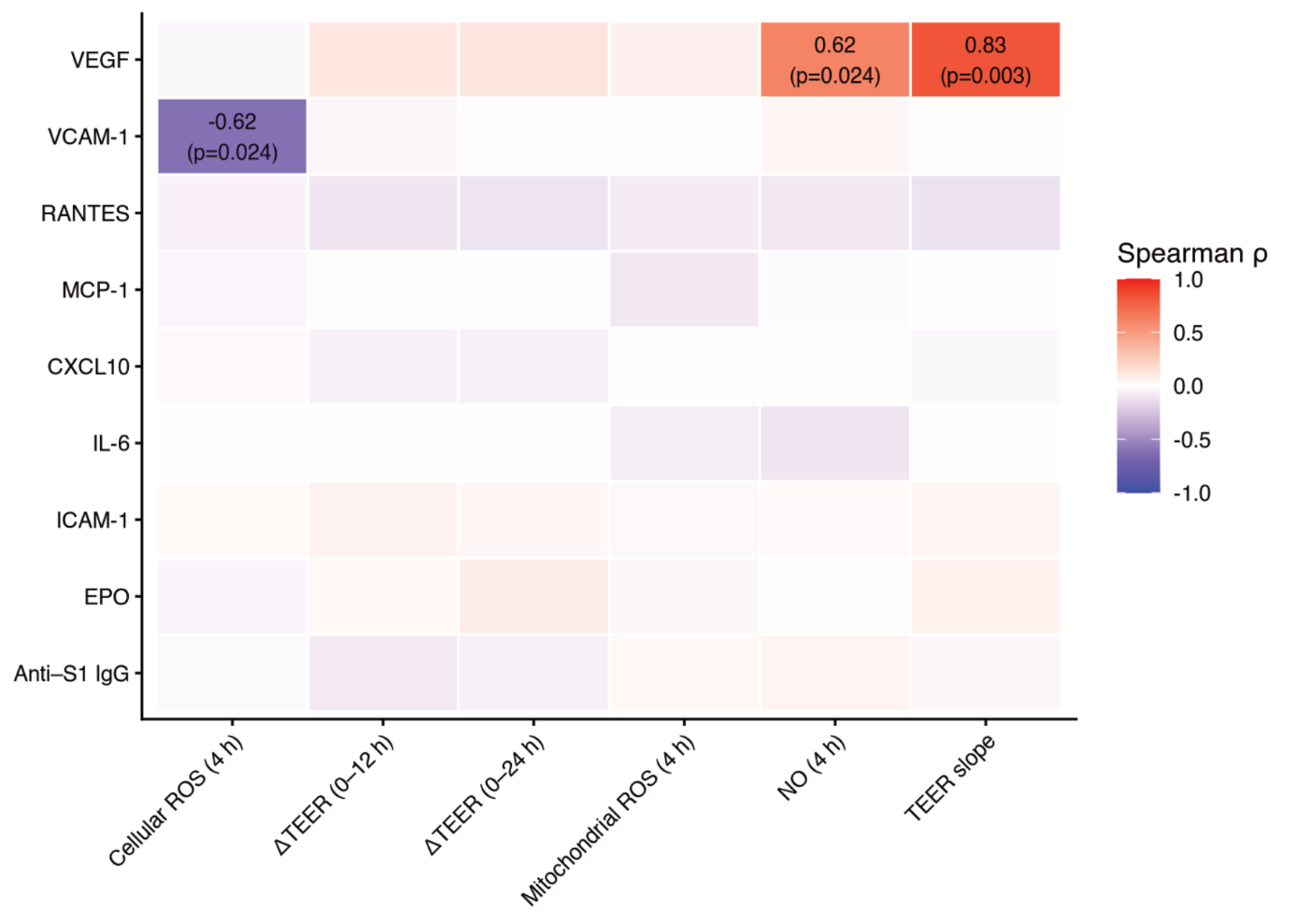


**Supplementary Figure 8. Exploratory correlations between plasma endothelial dysfunction markers and plasma-induced endothelial functional readouts.**
Heatmap depicting Spearman correlation coefficients (ρ) between circulating plasma markers related to endothelial dysfunction and inflammation (VEGF, IL-6, ICAM-1, VCAM-1, MCP-1, CXCL10, RANTES, erythropoietin [EPO], and anti-S1 IgG) and plasma-induced endothelial functional outcomes, including cellular and mitochondrial ROS, NO production, changes in TEER (ΔTEER), and TEER slope. Analyses were performed using pairwise complete observations. Color intensity reflects the direction and magnitude of Spearman’s ρ. Correlation coefficients and unadjusted p-values are shown for associations with p < 0.05. All analyses are exploratory and hypothesis-generating.

**Supplementary Tables**

**Supplementary Table 1: Primer pairs for real-time RT-qPCR**

| Gene Targets | Forward Sequence (3’-5’) | Reverse Sequence (5’-3’) | mRNA Accession Number |
| --- | --- | --- | --- |
| CXCL1 | ATCTGTCCCTGCCCTTCTTC | GACGACAGCAAAGATGACCC | NM_000634 |
| CXCL10 | GGTGAGAAGAGATGTCTGAATCC | GTCCATCCTTGGAAGCACTGCA | NM_001565 |
| ICAM-1 | AGCGGCTGACGTGTGCAGTAAT | TCTGAGACCTCTGGCTTCGTCA | NM_000201 |
| GAPDH | GTCTCCTCTGACTTCAACAGCG | ACCACCCTGTTGCTGTAGCCAA | NM_002046 |
| IL-6 | AGTGAGGAACAAGCCAGAGC | GTCAGGGGTGGTTATTGCAT | NM_000600 |
| IL-8 | GGTGCAGTTTTGCCAAGGAG | TTCCTTGGGGTCCAGACAGA | NM_000584 |
| MCP-1 | AGAATCACCAGCAGCAAGTGTCC | TCCTGAACCCACTTCTGCTTGG | NM_002982 |
| TNF | CTCTTCTGCCTGCTGCACTTTG | ATGGGCTACAGGCTTGTCACTC | NM_000594 |
| VEGF | TTGCCTTGCTGCTCTACCTCCA | GATGGCAGTAGCTGCGCTGATA | NM_001025366 |

Forward and reverse primer sequences and mRNA accession number for each gene target

| **Supplementary Table 2: Baseline characteristics of PCS and HC** | | | |
| --- | --- | --- | --- |
|  | **HC**  **n= 24** | **PCS patients**  **n=41** | ***p-***  ***value*** |
| **Age** | | | |
| Years, Mean (SD) | 44.6 (±12.2) | 42.2 (±12.2) | 0.44 |
| **Gender** | | | |
| female | 17 (70.9%) | 31 (75.6%) | 0.77 |
| **BMI** | | | |
| kg/m^2^, Mean (SD) | 23.2 (±3.6) | 24.2 (±3.9) | 0.33 |
| *Cardiovascular risk factors* | | | |
| **Current smoking** | 4 (16.7%) | 6 (19.4%) | 1.0 |
| **Art. hypertension** | 7 (29.2%) | 8 (25.8%) | 0.38 |
| *Acute SARS-CoV-2 Infection* | | | |
| **Variants** | | | |
| Alpha | - | 3 (7.3%) | - |
| Delta | - | 7 (17.1%) | - |
| Omicron | - | 10 (24.4%) | - |
| Unknown | - | 21 (51.2%) | - |
| **Clinical Progression Scale for COVID-19 severity (WHO)** |  |  |  |
| 0 | - | 0 (0%) | - |
| 2 | - | 24 (58.5%) | - |
| 3 | - | 13 (31.7%) | - |
| 4 | - | 2 (4.9%) | - |
| 5 | - | 1 (2.4%) | - |
| 6 | - | 1 (2.4%) | - |
| **Number of infections** | | | |
| 0 | 24 (100.0%) | 0 (0.0%) | - |
| 1 | - | 30 (73.2%) | - |
| 2 | - | 11 (26.8%) | - |
| **Number of vaccinations** |  |  |  |
| 0 | 24 (100.0%) | 3 (7.3%) | - |
| 2 | - | 15 (36.6%) | - |
| 3 | - | 23 (56.1%) | - |
| *PCS characteristics* | | | |
| **PCS duration**  Months, Median (IQR) - 10.0 (7.0—18.0) - | | | |
| **Work loss** | - | 8 (19.5%) | - |
| **Sick leave** | | | |
| Days, Median (IQR) | - | 122.0 (4.0 - 291.0) | - |
| *PCS symptoms & severity Scales* | | | |
| **ME/CFS** | - | 25 (60.9%) | - |
| **FSS** | | | |
| Median (IQR) | - | 6.0 (4.8 - 6.6) | - |
| **C19-YRS** | | | |
| Mean (SD) | - | 38.20 (±18.21) | - |
| **PCS Severity Score** | | | |
| Mean (SD) | - | 35.70 (±9.73) | - |
| **PHQ9** | | | |
| Mean (SD) | - | 10.72 (±4.47) | - |
| **Anti-S1 IgG** | | | |
| ng/mL, Median (IQR) | 385.5 (198.7 - 557.9) | 9049.7 (8538.5 - 9126.0) | **<0.001***** |
| *Standard laboratory values* | | | |
| **Leukocytes** | | | |
| Giga/L, Mean (SD) | 6.0 (±1.5) | 6.7 (±1.9) | 0.16 |
| **Haemoglobin** | | | |
| g/dL, Mean (SD) | 14.8 (±2.0) | 14.2 (±1.2) | 0.14 |

P-values are shown for statistical tests comparing post-COVID-19 syndrome (PCS) patients (n=41) with a healthy cohort (HC) (n=24). The Student's t-test was used for parametric variables, the χ2 test for categorical variables, and the Mann-Whitney U test for nonparametric variables. Clinical Progression Scale for COVID-19 severity: 0, Uninfected (no viral RNA detected); 2, Ambulatory – symptomatic, independent; 3, Ambulatory – symptomatic, assistance needed; 4, Hospitalized – no oxygen therapy; 5, Hospitalized – oxygen by mask or nasal prongs; 6, Hospitalized – non-invasive ventilation (NIV) or high-flow oxygen BMI; Body Mass Index, FSS; Fatigue Severity Scale; ME/CFS; Myalgic Encephalomyelitis/Chronic Fatigue Syndrome, C19-YRS; COVID-19 Yorkshire Rehabilitation Scale, Patient Health Questionnaire-9

**Supplementary Table 3: Regression models for potential confounders**

| Hb as the dependent value |  |  |  |  |
| --- | --- | --- | --- | --- |
| **Predictors** | ***CI*** | ***p-value*** | ***CI*** | ***p-value*** |
|  |  |  |  |  |
| **Anti-S1 IgG** | -0.00 – 0.00 | 0.836 | 0.00 – 0.00 | **0.034*** |
| Age |  |  | 0.02 – 0.03 | 0.601 |
| Gender, male |  |  | 1.88 – 3.11 | **<0.001***** |
| Arterial hypertension, y |  |  | 1.32 – 0.15 | 0.112 |
| Hypercholesterolemia, y |  |  | 0.17 – 1.07 | 0.15 |
| BMI |  |  | 0.05 – 0.11 | 0.506 |
| Nicotine abuse, y |  |  | 0.51 – 2.13 | **0.002**** |
|  |  |  |  |  |
| R^2^ / R^2^ adjusted | 0.001 / -0.027 |  | 0.734 / 0.670 | |

| Hct as the dependent value |  |  |  |  |
| --- | --- | --- | --- | --- |
| **Predictors** | ***CI*** | ***p-value*** | ***CI*** | ***p-value*** |
|  |  |  |  |  |
| **Anti-S1 IgG** | -0.00 – 0.00 | 0.598 | 0.00 – 0.00 | 0.191 |
| Age |  |  | 0.06 – 0.08 | 0.756 |
| Gender, male |  |  | 3.88 – 7.55 | **<0.001***** |
| Arterial hypertension, y |  |  | 3.54 – 0.86 | 0.223 |
| Hypercholesterolemia, y |  |  | 0.33 – 3.38 | 0.104 |
| BMI |  |  | 0.15 – 0.34 | 0.437 |
| Nicotine abuse, y |  |  | 0.53 – 5.40 | **0.019*** |
|  |  |  |  |  |
| R^2^ / R^2^ adjusted | 0.008 / -0.020 |  | 0.623 / 0.532 | |

| VEGF as the dependent value |  |  |  |  |
| --- | --- | --- | --- | --- |
| **Predictors** | ***CI*** | ***p-value*** | ***CI*** | ***p-value*** |
|  |  |  |  |  |
| **Anti-S1 IgG** | -0.00 – 0.00 | 0.222 | -0.00 – 0.00 | 0.247 |
| Age |  |  | -0.35 – 0.36 | 0.976 |
| Gender, male |  |  | 5.13 – 13.36 | 0.372 |
| Arterial hypertension, y |  |  | 6.96 – 15.00 | 0.461 |
| Hypercholesterolemia, y |  |  | 0.79 – 18.44 | 0.071 |
| BMI |  |  | -1.69 – 0.66 | 0.377 |
| Nicotine abuse, y |  |  | 9.23 – 13.57 | 0.701 |
|  |  |  |  |  |
| R^2^ / R^2^ adjusted | 0.038 / 0.013 |  | 0.178 /0.004 |  |

| Hb as the dependent value |  |  |  |  |
| --- | --- | --- | --- | --- |
| **Predictors** | ***CI*** | ***p-value*** | ***CI*** | ***p-value*** |
|  |  |  |  |  |
| **C19YRS** | -0.05 – -0.00 | **0.033*** | -0.03 – 0.00 | 0.081 |
| Age |  |  | -0.02 – 0.03 | 0.725 |
| Gender, male |  |  | 1.81– 3.06 | **<0.001***** |
| Arterial hypertension, y |  |  | -1.27 – 0.19 | 0.142 |
| Hypercholesterolemia, y |  |  | -0.01 – 1.29 | 0.054 |
| BMI |  |  | -0.07 – 0.08 | 0.863 |
| Nicotine abuse, y |  |  | 0.26 – 1.79 | **0.011*** |
|  |  |  |  |  |
| R^2^ / R^2^ adjusted | 0.127 / 0.102 |  | 0.765 /0.706 |  |

| Hct as the dependent value |  |  |  |  |
| --- | --- | --- | --- | --- |
| **Predictors** | ***CI*** | ***p-value*** | ***CI*** | ***p-value*** |
|  |  |  |  |  |
| **C19YRS** | -0.12 – -0.01 | **0.029*** | -0.09 – -0.00 | **0.03*** |
| Age |  |  | -0.06 – 0.07 | 0.897 |
| Gender, male |  |  | 3.76 – 7.28 | **<0.001***** |
| Arterial hypertension, y |  |  | -3.18 – 0.96 | 0.281 |
| Hypercholesterolemia, y |  |  | 0.43 – 4.09 | **0.017*** |
| BMI |  |  | -0.18 – 0.26 | 0.694 |
| Nicotine abuse, y |  |  | 0.11 – 4.42 | **0.04*** |
|  |  |  |  |  |
| R^2^ / R^2^ adjusted | 0.133 / 0.108 |  | 0.705 /0.631 |  |

| VEGF as the dependent value |  |  |  |  |
| --- | --- | --- | --- | --- |
| **Predictors** | **CI** | ***p-value*** | ***CI*** | ***p-value*** |
|  |  |  |  |  |
| **C19YRS** | -0.09 – 0.33 | 0.249 | -0.15 – 0.35 | 0.435 |
| Age |  |  | -0.32 – 0.42 | 0.796 |
| Gender, male |  |  | 4.67 – 15.67 | 0.279 |
| Arterial hypertension, y |  |  | 8.25 – 14.71 | 0.571 |
| Hypercholesterolemia, y |  |  | 3.28 – 17.92 | 0.169 |
| BMI |  |  | -1.81 – 0.49 | 0.253 |
| Nicotine abuse, y |  |  | 9.72 – 13.81 | 0.725 |
|  |  |  |  |  |
| R^2^ / R^2^ adjusted | 0.035 / 0.009 |  | 0.162 / -0.021 |  |

Regression models are shown for potential confounders of Haemoglobin (Hb), Haematocrit (Hct) and VEGF. Predictors include Anti-S1 IgG and the C19YRS severity score. In the multivariate model, a model was fitted for age, gender, obesity, arteriolar Hypertension, hypercholesterolemia, BMI, and nicotine abuse. F-statistic was significant for all shown significant models

**Supplementary Table 4: Baseline characteristics of PCS and HC used for cell culture experiments**

|  | **HC**  **n= 8** | **LC patients**  **n=13** | ***p-***  ***value*** |
| --- | --- | --- | --- |
| **Age** | | | |
| Years, Mean (SD) | 43.8 (±14.5) | 38.5 (±13.6) | 0.41 |
| **Gender** | | | |
| female | 6 (75%) | 10 (76.9%) | 1.00 |
| **BMI** | | | |
| kg/m^2^, Mean (SD) | 21.4 (±2.1) | 24.7 (±4.1) | 0.054 |
| **Obesity** | 1 (12.5%) | 4 (30.8%) | 0.61 |
| **Nicotine abuse** | 2 (25%) | 3 (23.0%) | 1.00 |
| **Art. hypertension** | 2 (25%) | 4 (30.8%) | 1.00 |
| **C19-YRS** | | | |
| Mean (SD) | - | 45.85 (±12.40) | - |
| **Leukocytes** | | | |
| Giga/L, Median (IQR) | 6.7 (5.5 - 7.9) | 6.2 (5.3 - 8.0) | 1.0 |
| **Haemoglobin** | | | |
| g/dL, Median (IQR) | 14.3 (13.3 - 14.9) | 14.2 (13.7 - 14.4) | 0.83 |
| P-values are shown for statistical tests comparing Post-COVID Syndrome (PCS) patients with a healthy cohort (HC), which were used for cell culture experiments. The Student's t-test was used for parametric variables, the χ2 test for categorical variables, the Mann‒Whitney U test for nonparametric variables, and the exact Fisher test for proportional variables. BMI; body mass index, C19-YRS; COVID-19 Yorkshire Rehabilitation Scale | | | |
